# Chromosome-level genome assembly and manually-curated proteome of model necrotroph *Parastagonospora nodorum* Sn15 reveals a genome-wide trove of candidate effector homologs, and redundancy of virulence-related functions within an accessory chromosome

**DOI:** 10.1186/s12864-021-07699-8

**Published:** 2021-05-25

**Authors:** Stefania Bertazzoni, Darcy A. B. Jones, Huyen T. Phan, Kar-Chun Tan, James K. Hane

**Affiliations:** 1 Centre for Crop & Disease Management, Curtin University, Perth, Australia; 2 Curtin Institute for Computation, Curtin University, Perth, Australia

## Abstract

**Background:**

The fungus *Parastagonospora nodorum* causes septoria nodorum blotch (SNB) of wheat (*Triticum aestivum*) and is a model species for necrotrophic plant pathogens. The genome assembly of reference isolate Sn15 was first reported in 2007. *P. nodorum* infection is promoted by its production of proteinaceous necrotrophic effectors, three of which are characterised – ToxA, Tox1 and Tox3.

**Results:**

A chromosome-scale genome assembly of *P. nodorum* Australian reference isolate Sn15, which combined long read sequencing, optical mapping and manual curation, produced 23 chromosomes with 21 chromosomes possessing both telomeres. New transcriptome data were combined with fungal-specific gene prediction techniques and manual curation to produce a high-quality predicted gene annotation dataset, which comprises 13,869 high confidence genes, and an additional 2534 lower confidence genes retained to assist pathogenicity effector discovery. Comparison to a panel of 31 internationally-sourced isolates identified multiple hotspots within the Sn15 genome for mutation or presence-absence variation, which was used to enhance subsequent effector prediction. Effector prediction resulted in 257 candidates, of which 98 higher-ranked candidates were selected for in-depth analysis and revealed a wealth of functions related to pathogenicity. Additionally, 11 out of the 98 candidates also exhibited orthology conservation patterns that suggested lateral gene transfer with other cereal-pathogenic fungal species. Analysis of the pan-genome indicated the smallest chromosome of 0.4 Mbp length to be an accessory chromosome (AC23). AC23 was notably absent from an avirulent isolate and is predominated by mutation hotspots with an increase in non-synonymous mutations relative to other chromosomes. Surprisingly, AC23 was deficient in effector candidates, but contained several predicted genes with redundant pathogenicity-related functions.

**Conclusions:**

We present an updated series of genomic resources for *P. nodorum* Sn15 – an important reference isolate and model necrotroph – with a comprehensive survey of its predicted pathogenicity content.

**Supplementary Information:**

The online version contains supplementary material available at 10.1186/s12864-021-07699-8.

## Background

The fungus *Parastagonospora nodorum* causes septoria nodorum blotch (SNB) of wheat (*Triticum aestivum*) and is a model species for necrotrophic plant pathogens. In order to provide insight on the evolutionary history and gene repertoire of this pathogen, a genome assembly of *Parastagonospora nodorum* model isolate Sn15 was first reported in 2007 [1]. This used Sanger shotgun sequencing of a genomic BAC library which produced a 37.5 Mbp draft genome reference with 108 scaffolds and 10,762 genes. It was the first species among the class Dothideomycetes for which a whole-genome reference was available [1] and has been used as a model species for cereal necrotrophs. This draft genome resource contributed to the discovery of three proteinaceous necrotrophic effectors (NEs) corresponding to known gene loci - ToxA [2], Tox1 [3] and Tox3 [4] - which are major host-specific virulence determinants in *P. nodorum*. The presence of additional NEs have been detected via their interaction with quantitative trait loci (QTL) corresponding to host sensitivity loci, but the genes encoding these effectors have not yet been identified [5–11] and others may have not yet been uncovered. In order to discover novel effectors in *P. nodorum* and in other fungal plant pathogens, it is important to ensure that the genome assembly and gene annotations are as accurate and reliable as possible.

Recent advances in long-read genome sequencing technologies, and established genetic and physical mapping techniques, have made whole-chromosome assembly of microbial genomes readily achievable [12–18]. Three decades ago, chromosome size and number estimates via pulsed-field gel electrophoresis (PFGE) of 11 *P. nodorum* isolates had estimated a range from 14 to 19, totalling 28 to 32 Mbp, ranging from 0.4 to 3.5 Mbp in length, with the smallest observed only in wheat and barley-infecting isolates [19]. The *P. nodorum* Sn15 genome assembly was progressively improved over subsequent years. It was updated in 2013 reducing the number of scaffolds from 108 to 91 [20], and again in 2016 with revised gene annotations that were supported by protein and transcriptome alignments and manual curation [21–23]. Leveraging these resources, comprehensive analyses of its genomic landscape and genome-based processes contributing to pathogenic adaptations have extended to transposable elements (TE) and gene repeats [1, 24, 25], repeat-induced point mutations (RIP) [24–26], mesosynteny [27], and multiple comparative genomics studies [14, 15, 23, 28, 29]. Initially, the Sn15 reference isolate was compared to a hyper-virulent isolate (Sn4) and a non-aggressive isolate (Sn79–1087) lacking known effector genes *ToxA*, *Tox3* and *Tox1* [20, 23]. These newly gained information and resources have played a vital role in studying important pathogenicity gene candidates. Subsequent comparison to an international panel (across 10 countries) of 22 *P. nodorum* and 10 *Parastagonospora avenae* isolates indicated presence-absence variation (PAV) - with notable absences in the ‘avirulent’ Sn79–1087 isolate assembly - of known effector loci and of large regions (i.e. scaffolds 44, 45, 46 and 51) [23], which was supplemented by a predictive analysis of accessory chromosome (AC) or region (AR) sequence properties (scaffolds 50 and 69) [29]. These large PAV regions were indicative of ACs/ARs that are associated with host-specific virulence in numerous fungal species [30], but this could not be confirmed with an unfinished genome assembly. In 2018, long-read-based genome assemblies were generated for 3 *P. nodorum* isolates (Sn4, Sn79–1087 and Sn2000) with 22 to 24 contigs [15]. Analysis of the Sn4 genome revealed that ‘contig23’ (~ 0.48 Mbp) was absent in Sn79–1087 and therefore considered an AC. This study also used transcriptome data from the Sn15 reference isolate to ‘auto-annotate’ genes in Sn4, and subsequently trained gene prediction software on the Sn4 annotations, which was used to perform in silico prediction of genes in the remaining isolates [15]. A follow up study in 2019 compared these four assemblies to NGS-based assemblies for a panel of 197 isolates from the United States, highlighted widespread diversifying selection within predicted effector loci and across the AC Sn4 contig23, reinforced the impact of the known ToxA, Tox1 and Tox3 effectors, and predicted 17 candidate effector loci with high levels of diversifying selection [31].

The recent updates to *P. nodorum* genomic resources enable consideration of the genomic landscape and sequence features which are relevant to pathogenicity or adaptation at the chromosome-scale, such as repeat-rich regions and mutation hotspots [26, 30, 32]. Long-read-based methods have significantly improved genome assembly of these previously challenging regions [12, 16, 18, 33, 34] and scaffold lengthscan be further improved with genome-finishing techniques including optical restriction [14, 35] or chromosome interaction mapping [36]. In fungal pathogens with “two-speed” genomes, repeat-rich regions typically accumulate mutations more rapidly than conserved gene-rich regions [26], leading to compartmentalisation of pathogen genomes into stable GC-equilibrated regions and AT-rich ‘mutation hotspots’, which can include pathogenicity-associated ACs or ARs [30]. For pathogenicity loci not residing within ACs, growing evidence supports their frequent location in sub-telomeric mutation hotspots [37–39] which may also be ARs. The segregation bias of certain gene functions to the sub-telomere may be associated with the role of heterochromatin found at sub-telomere region in regulating gene expression during infection [39] and protection of the core genome from interspersion of sub-telomeric heterochromatin [40].

The presence or absence of effector genes, or ACs/ARs that contain them, can determine host/cultivar-specific virulence for several pathogen species [30]. Bioinformatic methods for effector prediction are usually of a reductive nature, which filter the complete gene set down to an candidate effector subset based upon multiple criteria [41]. These methods typically require effector gene annotations not to have been missed in the complete gene set (at either assembly or gene prediction steps), and directly benefit from the proper application of transcriptome data to gene annotation, which for gene-dense genomes like those of fungi can pose a technical challenge [42]. In this study, we present an updated chromosome-scale genome assembly for *P. nodorum* reference isolate Sn15, combining long-read data and optical mapping to arrive at a near complete telomere-to-telomere assembly of 23 chromosomes. Sn15 gene annotations have also been updated integrating new transcriptome data and extensive manual curation, which will ensure its reliability and ongoing utility as a model necrotroph. Insights from comparative genomics analysis is presented for comparisons of the Sn15 reference isolate versus the Sn4, Sn2000 and Sn79–1087 long-read assemblies, and an international panel of NGS-based assemblies for 28 other *Parastagonospora* isolates. This has highlighted mutation hotspots and locational biases across the 23 chromosomes of Sn15, including a 0.4 Mbp accessory chromosome and several telomeric ARs. New effector gene predictions for Sn15 are also provided, integrating the wealth of past data for Sn15 with new data including PAV and diversifying selection across the international pan-genome. These aggregated resources for *P. nodorum* Sn15 will offer novel research opportunities and serve as a useful tool to enhance ongoing efforts to breed for crop disease resistance.

## Results

### A chromosome-level reference genome assembly for *P. nodorum* Sn15

In order to complete the Sn15 assembly, a combination of long read sequencing using PacBio technology and optical mapping were used. PacBio DNA sequencing generated 368,822 raw reads of 50 bp to 41 Kbp in length at ~71X coverage. Self-correction resulted in 118,028 corrected reads, totalling 1.31 Gbp with an average length of 10 Kbp. Corrected reads were assembled into a draft assembly of 36 gapless contigs ranging from 3.5 Mbp to 37 Kbp, with a total length of 37.4 Mbp at 33.6X coverage. Only 844 corrected reads (0.7%) were not assembled. One of the 36 contigs corresponded to the previously published mitochondrial DNA sequence [GenBank: EU053989] and was discarded. An optical map produced 23 maps with an estimated total length of 39.26 Mbp (Supplementary Text 1). Thirty out of the 35 assembled contigs (36.88 Mbp) aligned to the 23 optical maps. The 5 contigs that did not align were short (38 to 120 Kbp, or 0.84% of the contig assembly) and highly repetitive, with no predicted genes. The curated scaffolds of contigs aligned to the 23 optical maps - subsequently referred to as ‘chromosomes’- were numbered in descending size order based on the physical lengths predicted by the optical map (Supplementary Table 5). Fourteen chromosomes contained no gaps, and 8 gaps were added to join non-overlapping contigs within chromosomes 2, 3, 4, 6, 7, 10 and 20. Terminal ‘TTAGG’ tandem repeats indicating telomeres were observed at both ends of 21 chromosomes, with 2 having a single telomere. New repetitive regions comprised ~ 0.4% of the assembly. The new assembly had 286 fewer gaps than the previous version [20, 23] and there was a ~ 4 Kbp increase in the average length of AT-rich regions, a reduction of incompletely assembled AT-rich regions (− 46) and an increase in fully assembled AT-rich regions (+ 33) (Supplementary Table 3).

### A revised set of gene annotations aggregated from multiple sources of evidence, including new *in planta* RNA-seq, fungal-specific gene finding software and manual curation

**Fig. 1:**
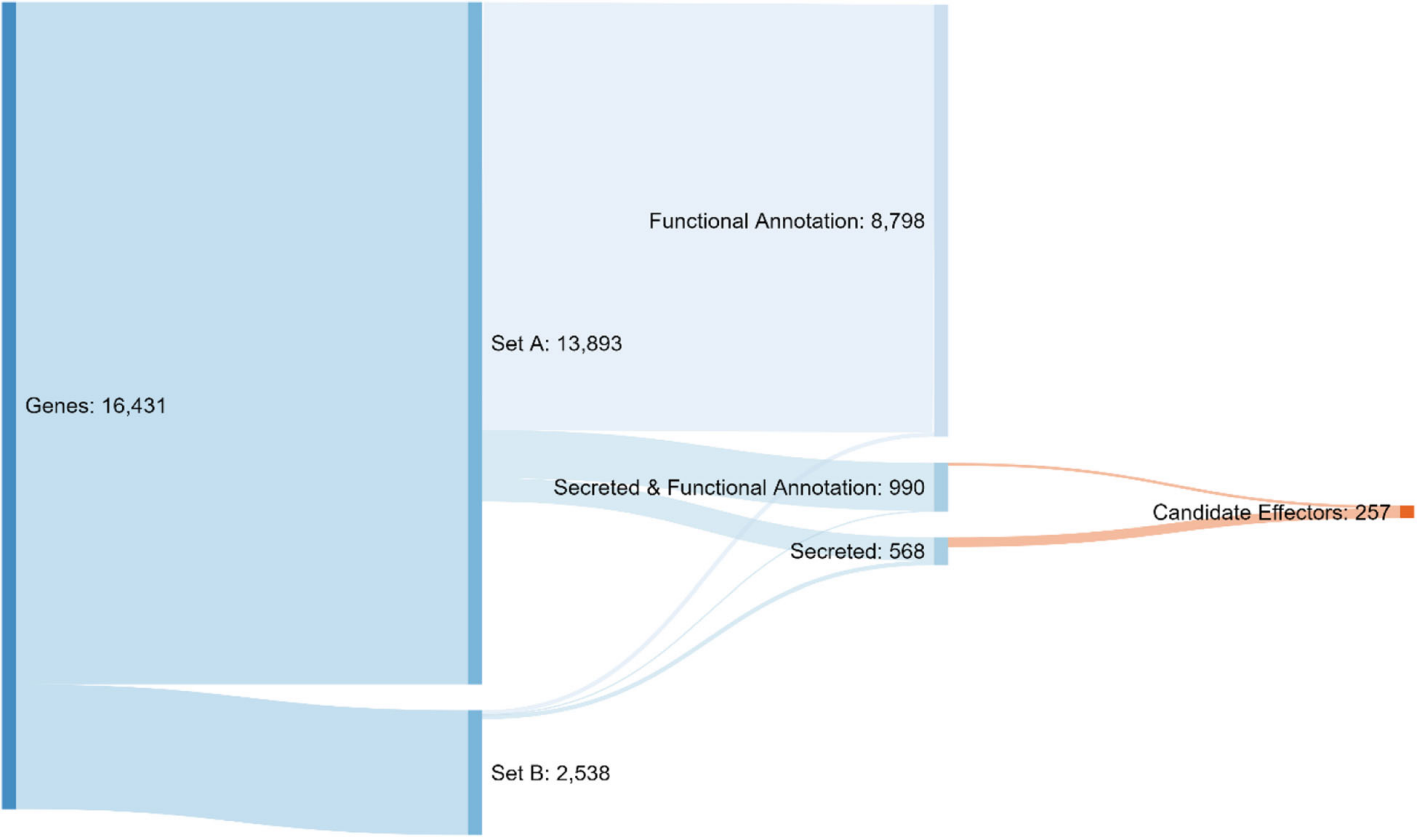
Summary of predicted genes of *Parastagonospora nodorum* Sn15. Predicted genes in Set A and B were separated based on predicted secretion signal peptides or informative functional annotations, from which effector candidates were predicted

**Fig. 2:**
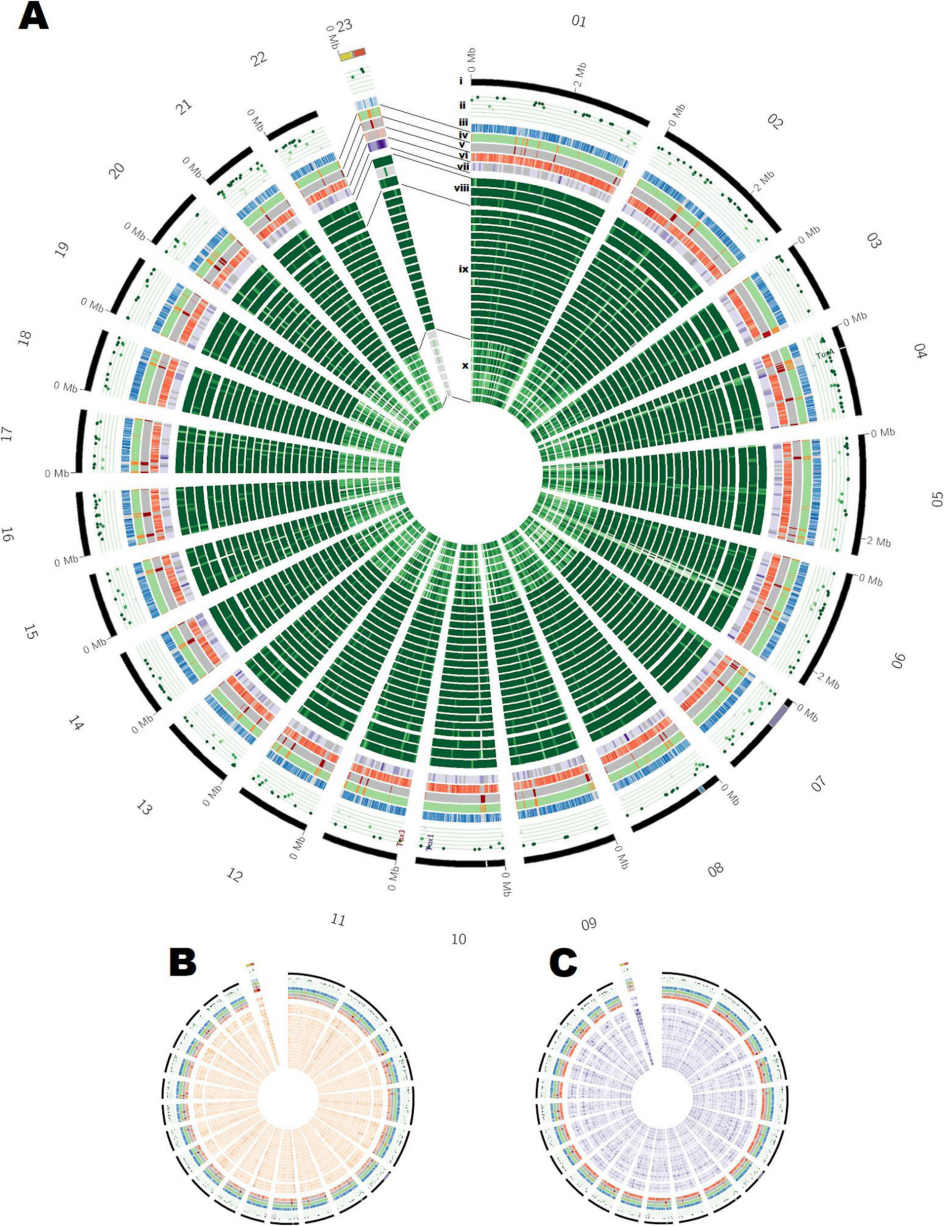
Sequence comparisons of the new genome assembly of the *Parastagonospora nodorum* Sn15 reference isolate with alternate *P. nodorum* isolates and *P. avenae* isolates, within 50 Kbp windows, for: **a** Presence-absence variation (PAV) indicated by percent coverage of MUMmer matches (green), **b** SNP density (red), and **c** the ratio of non-synonymous to synonymous SNP mutations (DN/DS) relative to Sn15 (purple). Rings indicate (in inwards order): i) Sn15 chromosome (black); ii) loci predicted by EffectorP and score from 0 to 1 (dark green); iii) gene presence (blue); iv) AT-rich regions (orange); v) repeat regions (red); vi) average SNP mutation density from (**b**) (orange); vii) average DN/DS from (**c**) (purple); viii) PAV versus alternate isolates Sn4, Sn79–1087 and Sn2000; ix) *P. nodorum* isolate draft assemblies; x) *P. avenae* isolate draft assemblies. *P. nodorum* Sn15 accessory chromosome 23 (AC23) has been highlighted with regions corresponding to scaffolds 44 (yellow) and 45 (red), previously reported in Syme et al. 2018 to be conditionally-dispensable and under positive selection

An estimate of the representation of the core gene content in the updated Sn15 assembly via BUSCO (v5.1.2) indicated 99.1% completeness versus the “fungi” dataset (fungi_odb10, 2020-09-10). The combination of various gene prediction methods (see methods), incorporating recently published in vitro and *in planta* RNA-seq data [23, 43]*,* fungal-specific gene prediction software, and manual curation, resulted in 16,431 predicted genes. This gene set was split into two subsets: a higher confidence set (Set A), and a lower confidence set to allow more sensitivity for subsequent pathogenicity gene predictions (Set B) (Fig. 1, Table 1A). Set A included 13,893 high confidence genes models with higher levels of support, whereas set B contained 2538 putative genes with either shorter coding sequence length or less RNA-seq support (Table 1B). Compared to the previously published annotation [20, 23], average gene length decreased by 70 bp and gene density increased by 2.8 genes per Mbp (Table 1A). Set B annotations were on average length 4 times shorter than those of Set A and in 86% of cases were a single exon (Table 1A). Of the 16,341 genes, 9788 were informatively functionally annotated (i.e. a conserved domain), and 990 of these also had a predicted secretion signal peptide (Fig. 1). The predicted secretome comprised 1568 genes of which 257 (1.5% of total genes and 25.3% of the secretome) were effector candidates (Fig. 1, Table 1). Across the Sn15 genome, gene density was inversely correlated with density of repetitive DNA (Fig. 2), with genes distributed at a relatively even density (~ 450 Mbp) except for accessory chromosome 23 (AC23 which was gene sparse (~ 380 Mbp) (Fig. 2). A lower proportion (36.7%) of loci were assigned functional annotations within AC23, which was 13% less than average. The known necrotrophic effector genes *ToxA*, *Tox1* and *Tox3* were all located within sub-telomeric regions of chromosome 4, 10 and 11 respectively, with *ToxA* also notably residing in the middle of a large (~ 570 Kbp) repeat-rich region (Fig. 2).

**Table 1:** Summary of new gene annotations of *P. nodorum* reference isolate Sn15. A) Comparison of high-confidence Set A and low confidence Set B to previous annotation versions and B) summary of data supporting gene annotations

**A) Summary**	**Previous studies** [20, 23]		**This study (Set A)**			**This study (Set B)**		
**Number of genes**	**13,569**		**13,869**			**2534**		
**Number of mRNAs**	**13,944**		**14,160**			**2557**		
**Average gene length, bp**	**1558**		**1488**			**373**		
**Number of exons**	**36,557**		**36,447**			**3070**		
**Average exons/gene**	**2.6**		**2.6**			**1.2**		
**Average exon length (bp)**	**556**		**539**			**299**		
**Gene density (genes / Mbp)**	**369**		**372**			**440 (Set A + Set B)**		
**B) Supporting Data**	**Loci**	**FPKM > 50**	**FPKM > 5**	**FPKM < 5**	**SignalP**	**SignalP+** **EffectorP**	**Region not in previous assembly**	**Fuctional annotation**
**Set A**	13,869	4033	9968	4030	1488	340	19	9505
In previous studies [20, 23]	13,663	3742	9633	4030	1463	323		9456
Not in previous studies [20, 23]	206	291	335	0	25	17		49
**Set B**	2534	220	2508	26	97	61	23	75
In previous study [1]	117	5	117	0	19	6		0
New or modified genes	2417	215	2391	26	77	55		75

### Comparative genomics

In comparisons of the Sn15 genome to alternate isolates, the Sn15 genome exhibited multiple large PAV regions (Fig. 2, Supplementary Table 6, Supplementary Table 7). Prior pan-genome and in silico studies using the previously published Sn15 assembly as its reference genome had indicated scaffolds 44, 45, 50 and 51 as regions of the genome with PAVs [20, 23, 29] (Supplementary Table 8).

Scaffold 50 corresponded to a sub-telomeric region of chromosome 8 (Supplementary Table 8). New reports of additional variable regions derived from this study include regions of chromosomes 7, 8 and 10. Chromosome 7 contained a ~ 455 Kbp region that is potentially duplicated in some isolates, but is represented in single copy in the current Sn15 assembly. Chromosomes 8 and 10 contained ~ 88 Kbp and ~ 10 Kbp PAV regions respectively. The PAV on chromosome 10 contained no genes, and the PAV on chromosome 8 contained 27 genes (Supplementary Table 9) but did not contain any predicted effector candidates (Supplementary Table 4).

Former scaffolds 44 and 45 corresponded to the ~ 444 Kbp AC23 of this study (Supplementary Table 8). Pan-genome alignment of Sn15 chromosomes with other *P. nodorum* and *P. avenae* isolates indicated that chromosome 23 was absent in *P. avenae* and the non-aggresive *P. nodorum* isolate Sn79–1087 (Fig. 2), which suggested that it lacked genes required for viability and was an accessory chromosome. In contrast, the majority of other “core” chromosomes were well conserved across *Parastagonospora* spp. AC23 also exhibited higher overall levels of non-synonymous mutations indicating diversification across this population relative to the Sn15 reference isolate (Supplementary Table 10). However the mutation profile of AC23 contained two regions separated by a large repeat island – each side corresponding to scaffolds 44 and 45 of the previously Sn15 assembly [20, 23] - which exhibited distinctly different mutation rates (Fig. 2). Comparison of AC23 to other Sn15 chromosomes did not indicate that it had originated from duplication of core chromosomes (Supplementary Figure 1), however homologous (non-repetitive) regions in *Pyrenophora tritici repentis* [14, 44] and *Bipolaris* spp. [28, 29] genomes tended to be located in sub-telomeric regions (Supplementary Figure 2).

**Fig. 3:**
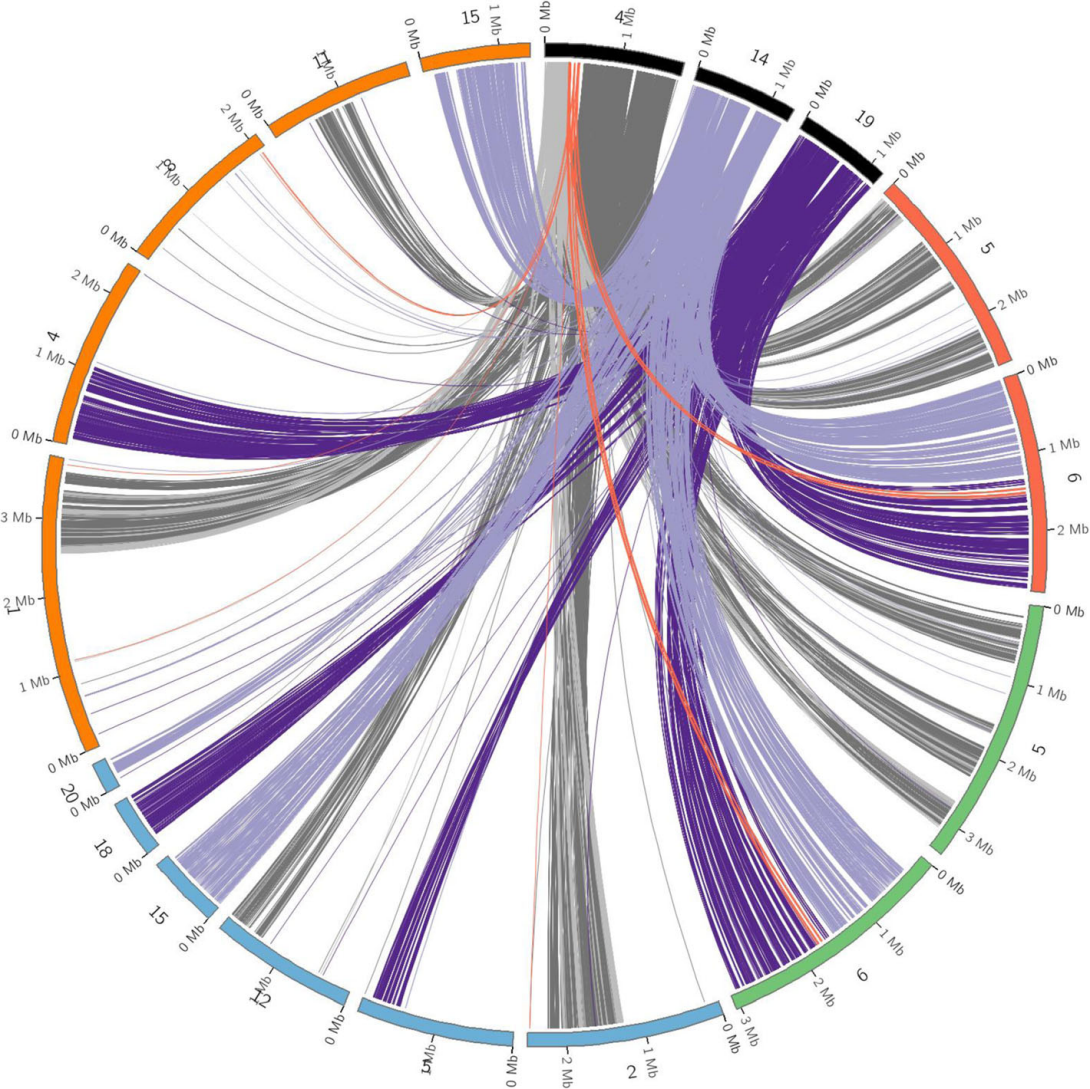
Sequence similarity comparisons between *ToxA*-containing and related sequences of (A, black) *P. nodorum* Sn15 (chromosomes 4, 14 and 19); (B, red) *Pyrenophora tritici-repentis* BFP (chromosomes 5 and 6); (C, green) *Pyrenophora tritici-repentis* M4 (chromosomes 5 and 6); *Bipolaris maydis* (blue) (scaffolds 2, 5, 12, 15, 18 and 20); and *Bipolaris sorokiniana* CS10 (orange) (chromosomes 1, 4, 8, 1 and 15). Matches with *P. nodorum* Sn15 chromosome 4 are coloured grey, with the *ToxA*-containing region highlighted in red, and matches with *P. nodorum* Sn15 chromosomes 14 and 19 and coloured light and dark purple respectively

The previous scaffold 51 corresponded to a ~ 74 kbp region within a repeat-rich sub-telomeric region of chromosome 4 (Supplementary Table 8), which also contained the effector gene *ToxA*. This sub-telomeric region had below average GC content, correspondingly high repeat content (~ 18.3% higher than the genome average), increased mutation density and less than half of the average gene density (Supplementary Table 6). The 9 predicted loci within this region had an average DN/DS of 1.9, more than double the genome average (Supplementary Table 6). Alignment of this region between the Sn15 assembly presented in this study, and the long read assemblies of Sn4, Sn2000 and Sn79–1087, showed structural variations that may indicate that breakage-fusion bridge (BFB)-mediated rearrangements (distal translocations between chromosomes lacking telomere caps) may have occurred in one or more of these isolates (Supplementary Figure 3) [45]. Comparisons of this region to corresponding regions containing ToxA homologs in related species *Pyrenophora tritici-repentis* [14, 44], *Bipolaris maydis* [29] and *B. sorokiniana* [28], indicated further chromosome structure diversity. The sub-telomeric *ToxA* region of chromosome 4 in *P. nodorum* appeared to be consistent with *Bipolaris* spp. where it was also found in sub-telomeric locations. In contrast, the *P. tritici-repentis ToxA* -containing chromosome appeared to be a product of the breakage of the *P. nodorum ToxA* region, followed by chromosome fusions resulting this region being flanked by sequences corresponding to *P. nodorum* chromosomes 14 and 19 (Fig. 3).

## Discussion

### The chromosome-level assembly for *P. nodorum* reference isolate Sn15 improved detection of pathogenicity gene-rich regions

The new chromosome-level genome assembly of *P. nodorum* Sn15 created by this study has established the correct number of chromosomes for this pathogen, which was previously underestimated by PFGE to range from 14 to 19 [19], and is consistent with 22–23 observed in assemblies of other isolates [15, 31]. PFGE fragment resolution accuracy requires at least ~ 1% difference in chromosome size [46], meaning 6 out of the 23 assembled sequences were within a potentially unresolvable size range (Supplementary Table 5). The difference in chromosome number between the two studies therefore is justified. This study also presents 21 out of 23 chromosomes with both telomeric ends and 14 gapless chromosomes. In addition, the new chromosome-level genome assembly for Sn15 was also supported by transcriptome data and manually curated gene annotations, and related bioinformatic resources for the *P. nodorum* Sn15 reference isolate were updated, enhancing these important resource for studying molecular host-pathogen interactions and for effector discovery [41, 47, 48].

Chromosome-level analysis of the genomic landscape can enable detection of compartmentalised mutation ‘hot spots’ that may contain pathogenicity loci - a commonly reported feature for “two-speed” genomes which have been broadly affected by transposon activity and repeat-induced point mutation (RIP) [24, 26]. As genome assemblies have been improved towards chromosome-scale representation, there have also been several reports of pathogenicity genes within sub-telomeric locations in other pathosystems [15, 17, 37, 38, 40]. Thus, the new Sn15 assembly presented new opportunities to predict novel pathogenicity-related genes within the ‘two-speed’ regions that are repeat-rich or conditionally-dispensable [26, 30, 32]. Presumably, the apparent bias of pathogenicity gene locations within mini-chromosome or sub-telomeric regions could be associated with BFB formation [45] and mesosyntenic rearrangements [27, 32] between chromosome termini. Indeed, pan-genome comparisons indicated that the 0.4 Mbp AC23 was an accessory chromosome, with gene content relevant to pathogenicity (see below). The updated Sn15 assembly also highlighted additional regions not present in previous assembly versions, which comprised ~ 152 Kbp of repetitive DNA (0.4% of the genome) and 86,468 bp of non-repetitive DNA. While these represented a very small proportion of the genome, they may have special significance for plant pathology as they are more likely to contain effector or other pathogenicity genes. The relative placement of these regions in the genomic landscape was also important in assessing their likely roles in pathogenicity adaptation [26, 30, 32] as a parameter for effector prediction [41].

### Candidate effector genes were derived from extensive gene annotation data for *P. nodorum* Sn15

Considerable efforts have been made across previous studies to ensure the reliability and ongoing applicability to plant pathology research of the annotated gene set for *P. nodorum* Sn15 [1, 20–23, 42], particularly for the purpose of effector and pathogenicity gene discovery. The revised Sn15 gene set includes a primary set of 13,893 genes (Set A) and a lower confidence set of 2538 (Set B) which was retained to enhance the sensitivity and capacity of effector gene predictions. The total number of predicted genes has increased since the previous annotation version [23], and is also higher than the currently reported average across the Ascomycota [49]. However we note general trends across all species, that while reducing assembly fragmentation can reduce the total number of predicted genes [50], the addition of significantly improved transcriptome data [51] or gene prediction methods [42] can increase this number. Functional annotations were assigned to 59.5% of predicted genes (Set A + B, excluding non-specific features e.g. coiled-coils, intrinsic disorder).

Across the whole genome, 257 effector candidate genes were predicted (Fig. 1, Supplementary Table 4), a number comparable to similar fungal pathogen genome surveys [41]. Effector candidate genes exhibited the typical features expected of effectors, including: secretion, low molecular weight, cysteine richness, diversifying selection, association with mutation hotspots, and where functional annotations were assigned these had a common pathogenicity-related theme (Supplementary Table 4). Secretion was predicted for 1558 genes (9.5% of Set A + B), of which 257 (16.5% of predicted secretome) were effector candidates and 12 were predicted to localise to the chloroplast (including the confirmed effector ToxA) (Supplementary Table 4). Effector candidate loci were typically found within either 5–10 or 20–25 Kbp of AT-rich regions, which was not the case across the whole gene set (Supplementary Table 4, Supplementary Table 11). This is consistent with reports of RIP and effector location bias within AT-rich mutation hotspots [26]. The *ToxA* locus was 4039 bp and *Tox3* was 1860 bp from their nearest respective AT-rich regions. The *Tox1* locus was located > 200 Kbp from its nearest AT-rich region, however all 3 effector loci were also located within sub-telomeric regions (Fig. 2). This association between telomeres and effector-rich mutation hotspots is also reported in other pathogen species [30, 32]. Comparison of orthologs between the Australian reference isolate Sn15 and the US isolate Sn4 [15], indicated 14 out of the 17 previously published Sn4 candidate effectors were also predicted among the Sn15 candidates (Supplementary Table 12). These Sn4 candidates – which included *Tox1 -* were previously reported to exhibit diversifying selection that was specific to one of the 2 major US sub-populations [31].

### Functionally-redundant genes may be associated with potential pathogenic properties of accessory chromosome 23

Surprisingly, AC23 which exhibited typical characteristics of ACs [15, 30, 31, 52] - and may correspond to anecdotal reports of a ~ 0.4 Mbp AC specific to *P. nodorum* wheat and barley-infecting isolates [53] - had a relatively low density of effector candidate loci (Supplementary Table 4, Supplementary Table 10). Six candidate effector loci were predicted on AC23 in a previous study [23], with two of these (SNOG_16226 and SNOG_16236) re-predicted (with ranked scores of 10 and 9 respectively) in the more stringent predictions of this study. AC23 also encoded multiple genes with other pathogenicity-related and/or redundant functions, which may indicate tandem duplications or multiple BFB events. These functions included: Ulp1 protease (SNOG_16274, SNOG_16214), RING/FYVE/PHD-type zinc finger proteins (SNOG_16310, SNOG_16333), valyl-trna synthase (SNOG_16268, SNOG_16213, SNOG_16211), and UstYa-like protein (mycotoxin biosynthesis) (SNOG_16357) (Supplementary Table 13). Ulp1 protease is involved in the modification of SMT3, a ubiquitin-like protein of the SUMO family which suppresses MIF2 mutations. MIF2 is a centromere protein that regulates stability of di-centromeric mini-chromosomes in baker’s yeast [54]. Its presence on AC23 is notable given that AC23 is a mini-chromosome and is therefore more likely to be unstable. UstYa-like proteins are involved in the secondary metabolite synthesis of cyclic peptide mycotoxins including ustiloxin and chyclochlorotine [55], however the products of many remain unknown. FYVE domain zinc finger proteins reportedly may bind to phospholipid PI3P [56], which could potentially facilitate host cell uptake.. The genes and functions listed above represent candidate pathogenicity loci residing on AC23 which are of high importance for further investigation.

### A trove of effector and pathogenicity gene homologs were predicted among candidate effector-loci

We previously observed that the deletion of *ToxA*, *1* and *3* in *P. nodorum* SN15 resulted in a mutant that retained near-WT level of virulence on most commercially adopted wheat varieties [57, 58]. This suggested that SN15 that lacked *ToxA*, *Tox1* and *Tox3* may have produced undiscovered effectors or other virulence factors to functionally compensated for the loss of these major NE genes [59, 60]. In addition, biochemical and genetic characterisation of US *P. nodorum* isolates identified evidence of other NEs [5–11]. This prompted us to apply a bioinformatic approach to predict for NE candidates in the near-complete SN15 genome that are relevant to the Australian cereal industry. From the prediction analysis, *ToxA*, *Tox1* and *Tox3* ranked highly among the top 98 Sn15 candidates with ranked scores of 5 and above (Supplementary Table 10). While remaining candidates are unconfirmed, among these we observed a wealth of assigned functions or matches strongly suggesting roles in pathogenicity. *SNOG_13622* and *SNOG 08876* encode for CFEM domain proteins, which have roles in iron acquisition and several of which have been reported with roles in virulence [61]. *SNOG_42372* and *SNOG_07772* encode for chitin-binding LysM domain proteins which offer protection from PTI in the host [62]. *SNOG_07596* encodes a thaumatin-like protein, which when produced by host plants are pathogenesis-related (PR) proteins involved in defence, however fungal homologs have also been reported with roles in virulence [63]. *SNOG_03746* encodes a knottin-like protein. Knottins are cytotoxins that are best represented by snake and arachnid venoms, with the first fungal report of a knottin in the poplar rust *Melampsora larici-populina* [64]. *SNOG_30910* encodes a homolog to phospholipase A2 - which cleaves sn-2 acyl bond between 2 phospholipids that releases arachidonic acid and lysophosphatidic acid - and is also a common domain in spider, insect and snake venoms that disrupt cell membranes [65]. *SNOG_00200* encodes a product similar to *Alternaria alternata* allergen 1 (AA1-like). The AA1-like family [66] contains the *V. dahliae* effector PevD1, which binds the host thaumatin PR5 [67]. *SNOG_00182*, *SNOG_02182*, and *SNOG_16063* encode ribotoxins, which have a conserved sarcin/ricin loop (SRL) structure that cleaves specific sequences in the host rRNA, leading to ribosome inactivation and cell death by apoptosis [68]. *SNOG_13722* encodes a cerato-platanin, which induces phytoalexin synthesis and causes necrosis [69]. *SNOG_06012* encodes a protein similar to gamma crystallin/yeast killer toxin, which is a pore-forming cytotoxin [70]. *SNOG_01218* encodes a subtilisin, a serine protease family that is frequently reported in fungi to promote virulence [71]. *SNOG_03959* encodes a protein similar to a cyclophillin-like/peptidyl-prolyl cis-trans isolmerase (PPIase) [72], which in humans is well known for interfering with the immunosuppressive drug cyclosporin A, but is widespread across eukaryotes and has been reported as virulence determinants in several fungi including: *Leptosphaeria* spp., *Botrytis cinerea*, *Cryphonectria parasitica*, *Puccinia triticina*, *M. oryzae*, and *Lhellinus sulphurascens*, as well as various oomycete species of the *Phytophthora* genus [73]. *SNOG_08289* encodes a pectin/pectate lyase, which are reported in many fungi to promote virulence [74]. *SNOG_11034* encodes a protein similar to Egh16, an appressorially-located virulence factor of *Blumeria graminis* f. sp. *hordei* with broadly conserved homologs across several pathogenic fungal species [75]. *SNOG_15608* encodes a cutinase, which may be involved in host surface penetration [76]. *SNOG_02399, SNOG_03334, SNOG_40970, SNOG_08150, SNOG_04779* all encode for proteins with lipid interacting domains. *SNOG_11842* encodes a Hce2 effector homolog - which is named after Homologs of *C. fulvum* ECP2, a necrosis inducing effector. There are 3 defined classes of proteins with Hce2 domains, of which SNOG_11842 belongs to class I, the smallest and most common class [77]. Many of the above candidates with pathogenicity-related functional annotations are also expressed higher *in planta* (IP) relative to in vitro (IV) by a factor of 5, however the *Tox3* IP:IV is only 2 indicating this lower values may also be relevant in host-pathogen interactions. A lower-ranked candidate (*SNOG_06459*) with a ranked score below 5 is also mentioned here as it encoded a cerato-ulmin homolog. Cerato-ulmin is a hydrophobin, which is not a functional class normally reported to be directly involved in pathogenicity, but has been reported as a potential virulence factor in dutch elm disease [78]. Its mode of action is not like a typical effector however, as its role appears to be to protect spores from desiccation, which leads to increased spore survivability and transmission.

### Multiple effector candidate loci were predicted to be laterally-transferred with other cereal-pathogenic fungal species

Of the 98 highly-ranked effector candidates, 11 showed a conservation pattern indicating potential lateral transfer when compared to a panel of whole gene sets of > 150 fungal species (Supplementary Table 4) [79]. This included *SNOG_16571* (*ToxA*), *SNOG_20078* (*Tox1*), *SNOG_13622* (CFEM domain)*, SNOG_15952* (ribotoxin-like), *SNOG_00152, SNOG_01658, SNOG_20100, SNOG_08426, SNOG_07039, SNOG_00726,* and *SNOG_14618*. These had rare orthology relationships indicating potential lateral gene transfer (LGT) with *Pyrenophora* spp., *Setosphaeria turcica*, *Alternaria brassicicola*, *Verticillium dahliae*, *Leptospaheria maculans* and *Coccidioides immitus*. Aside from *SNOG_13622*, *SNOG_15952*, and known effectors *ToxA* and *Tox1*, this group of effector candidates had no predicted functional annotations. As expected, *SNOG_08981* (*Tox3*) was not included in this set and has so far been reported to have no known homologs.

## Conclusions

The *P. nodorum* isolate Sn15 was the first representative of the class Dothideomycetes with a genomic survey report [1], and has since become an important reference and model necrotroph with a significant set of accumulated genomic, transcriptomic, proteomic and bioinformatic resources supporting its genome and gene data [15, 20–25, 31, 43]. This study updates these resources in the context of a chromosome-scale assembly, identifying genome features relevant to pathogenicity i.e. sub-telomeric regions, accessory chromosomes and mutations hotspots. This has provided genomic context to subsequent predictions of candidate genes encoding effectors and other pathogenicity factors. In contrast to the earliest Sn15 genome study, effector candidates were supported by a wealth of functional annotation and comparative genomics data indicating strong homology to known effectors and other pathogenicity genes. This study is an important step forward for the further characterisation of *P. nodorum* chromosome structure and its role in pathogenicity, particularly in highly mutable and potentially effector-rich regions of the genome including AC23. Additionally, the increased representation of repeat-rich regions and provision of curated gene annotations within them, is of high value to ongoing efforts to characterise and understand fungal effectors. We anticipate future studies will utilise the effector predictions provided in this study to confirm new novel *P. nodorum* effectors, and potentially discover a role for AC23 in promoting virulence.

## Methods

### Genome sequencing and assembly

Genomic DNA of *P.nodorum (syn. Phaeosphaeria nodorum, Stagonospora nodorum, Leptosphaeria.*

*nodorum,. Septoria nodorum*) strain Sn15 [20] – originally isolated in Western Australia by the Dept. Primary Industries and Regional Development (DPIRD: Agriculture & Food) - was sequenced via Pacific Biosciences P5-C3 chemistry with 4 SMRT cells, at the Génome Québec Innovation Centre (McGill University, Montreal, QC, Canada). The longest 25% of reads were self-corrected and assembled using Canu v1.0 (−pacbio-raw, expected genome size 39 Mbp) [13]. Assembly base-calls were corrected with Pilon v1.16 [80] using Illumina reads [20] which were mapped to the assembly with Bowtie2 v2.3.3.1 [81]. Mitochondrial contigs assembled by the above methods were identical to a previously published Sn15 mtDNA [GenBank: EU053989] [1] therefore the old mtDNA record was not updated by this new assembly..

Optical maps were used to order and orient the Canu-assembled Sn15 contigs into a complete genome. Sn15 protoplasts were extracted from hyphae as per Solomon et al [82], which was adjusted to 1x10e8 with GMB (0.125 M EDTA pH 8, 0.9 M sorbitol) at 42 °C. Protoplasts were added 1:1 to 1% low melt agarose (SeaPlaque GTG in 2% sorbitol and 50 mM EDTA) and poured into Plug Mold (Bio-Rad Laboratories, Munich, Germany) and set at 4 °C for 30 min. The plug was added to 5 ml Proteinase K solution (1 mg/ml Proteinase K, 100 mM EDTA pH 8.0, 0.2% Na deoxycholate, 10 mM Tris pH 8.0 and 1% N-lauroyl sarcosine) and incubated at 50 °C overnight, then added to sterile wash buffer (20 mM Tris pH 8, 50 mM EDTA pH 8) for 4 h changing the solution every hour. Clean plugs were transferred into 0.5 M EDTA at 9.5 pH and stored at 4 °C until shipment at room temperature. High molecular weight DNA was extracted from protoplasts as per Syme et al [23] and digested with *Spe*I, resulting in 63,440 fragments with an average size of ~ 315 Kbp. Optical maps were generated and manually curated with MapSolver™ (OpGen, MD, USA). Contig joins were made by inserting a 100 bp unknown (N) gap. Where the optical map indicated contig mis-assemblies, potential breakpoints were inspected for a localised drop in aligned read coverage. Chromosome scaffolds were numbered in descending size order based on the estimated physical lengths derived from the optical map. Chromosome scaffolds were assessed by Quast v5.2 [83], BUSCO 5.1.2 [84] and by coverage depth of alignments of raw and corrected SMRT reads by bwa-mem (0.7.17-r1188, −x pacbio) [85] via SAMtools [86] and BEDtools [87]. The assembled Sn15 chromosome scaffolds of this study were compared to previously published assembly versions (Supplementary Table 1) [15, 23] with MUMmer 3.0 (nucmer -maxmatch, show-cords) [88] and alignments were visualised with Dot [89]. The SN15 reference genome data is available under BioProject: PRJNA686477. The updated SN15 genome assembly is deposited under [Genome: GCA_016801405.1/ASM1680140v1] and [NUC: CP069023.1 - CP069045.1].

### Annotation of genome features

Repetitive DNA regions within the Sn15 genome assembly were analysed by three methods. The presence and overall proportion of AT-rich regions were calculated with OcculterCut v1.1 [26]. Annotation of repeat regions was performed using RepeatMasker 4.0.6 (sensitive mode, rmblastn version 2.2.27+) [90] in four separate analyses, using: a) a published set of de novo repeats derived from a previous Sn15 genome assembly [24], b) RepBase (taxon “Fungi”) [91], c) LTRharvest of the GenomeTools suite [92], and d) a newly predicted set of de novo repeats generated by RepeatModeler v1.0.8 [93] (−engine ncbi -pa 15). Subsequent repeat analyses requiring a repeat-masked input used the output derived from the new de novo repeat dataset (Supplementary Table 2, Supplementary Table 3). Tandem repeats were predicted using Tandem Repeat Finder (Parameters: Match = 2, Mismatch = 7, Delta = 7, PM = 80, PI = 10, Minscore = 180, MaxPeriod = 2000) [94]. Telomere regions were identified by terminal “TTAGGG” tandem repeats [95].

Protein-coding gene loci were annotated incorporating multiple transcriptome datasets from previous studies [23, 43]. RNA-seq reads from a prior study [43] were trimmed with cutadapt v.9.1 (paired end mode, −-quality-cutoff = 30, −-minimum-length 25, −n 3) [96] and de-duplicated with khmer v2.0 and screed v0.9 (normalize-by-median.py, −C 30 -M 100e9) [97, 98]. Fungal RNA-seq reads were derived from a mixed *in planta* library during early infection (3 dpi) when known effectors are maximally expressed [60]. Fungal reads were separated from wheat sequences with BBsplit v36.11 (BBmap, Seal v36.11, −Xmx200g) [99] to screen against the *Ttriticum aestivum* assembly TGAC v1.30 (GCA_900067645.1) [100]. Filtered reads were mapped to the new Sn15 assembly with STAR v2.5.2b (alignReads, −-outSAMstrandField intronMotif --outFilterIntronMotifs) [101]. Transcript assembly was performed with Trinity v2.2.0 (−-seqType fa --trinity_complete --full_cleanup --jaccard_clip) [102]. RNAseq reads were aligned to the SN15 assembly with TopHat v2.2.6.0 (defaults) [103]. Relative expression was quantified with Cufflinks [104] and Stringtie v1.3.3b (params -m 50 -B -e -p 8) [105].

A final set of gene annotations was generated by combining annotations from multiple sources. Initial transcriptome-based predictions were made with PASA v2.0.2 [106], incorporating: Trinity transcripts; open-reading frames generated with Transdecoder v2.0.1 [102]; CodingQuarry (CQ) v2.0 predictions based on TopHat outputs [42]; a second round of predictions generated using Coding-Quarry “Pathogen Mode” (CQPM) (*A. testa*, 2016) within regions between the initial CQ predictions. De novo prediction was performed with GeneMark-ES v4.32 (−-ES, −-fungus) [107]. Previously published gene annotations for Sn15 were aligned to the new assembly with AAT r03052011 [108] using CDS features (−-dds ‘-f 100 -i 20 -o 75 -p 70 -a 2000’ --filter ‘-c 10’ --gap2 ‘-× 1’) and protein sequences (−-dps ‘-f 100 -i 30 -a 200’ --filter ‘-c 10’ --nap ‘-× 10’). All predicted gene annotations described above were assigned relative weight scores (AAT protein mapping 1, EST 5, AAT CDS mapping, GeneMark-ES 1, CQ/CQPM 10, transdecoder 10, PASA 9) and were then integrated into a single annotation set via EvidenceModeler (EVM) v1.1.1 [106]. Every locus of the EVM annotation set was manually curated using Webapollo [109] alongside supporting evidence from the various prediction methods described above, InterProScan domains aligned to the genome [110], aligned RNAseq reads [23, 43] and annotated repeat features (see above). New loci were manually annotated within intergenic regions if supported by RNAseq alignments. The resulting set of manually curated annotations were filtered (Table 2) for either: 1) orthologous best hit to the 13,690 gene models from the previously published annotation [20, 23] or; 2) coding regions (CDS) of > 300 bp in length and with RNAseq read coverage of > 50 fragments per kilobase of transcripts per million mapped reads (FPKM). This filtered set of manually curated genes is subsequently referred to as the primary gene set (Set A). The remaining predictions that failed this filter were retained as a secondary gene set (Set B) if CDS length was > 90 bp and RNAseq depth was > 5 FPKM or if homologous to a previously annotated gene [1, 20, 23]. To be consistent with previous publications on *P. nodorum* genomics, in this study gene annotations corresponding to loci that had been numbered in previous studies [1, 20, 23] retained their previous locus number despite non-sequential order along the new assembly. New annotations not corresponding to previously annotated loci were numbered from SNOG_40000 onwards.

**Table 2:** Criteria used to predict the primary gene prediction set (Set A) for P. nodorum Sn15, and the secondary set (Set B) which contains low-confidence gene predictions for the purpose of extracting a small proportion of strong effector candidates

Criteria	Set A (high confidence)	Set B (putative)
CDS length Gene expression Orthology/Homology	> 300 bp / > 100 aa *AND* > 50 FPKM *OR* Orthology (reciprocal best hit) to previous annotation [20, 23]	(> 90 bp / > 30 aa *AND* > 5 FPKM) *OR* Homology to previous annotation set [20, 23]

Various software and databases were used to assign functional annotations for both gene sets (A and B). OcculterCut v1.1 [26] predicted AT-rich regions and distances to the nearest AT-rich region for each locus. InterProScan (5.27–66.0) [110] was used to generate a broad range of functional annotations (InterPro, Pfam, Gene3D, Superfamily, MobiDB). PHIbase v4.2 [48] was searched to assign homology to known effectors. SignalP v4 [111]. was used to predict extracellular secretion, EffectorP v2.0 [47] was used to predict effector functions, and Localizer v1.0 (−e mode) [112] was used to predict potential host-cell sub-cellular localisation. The dbCAN r07/20/2017 [113] and AntiFam r3.0 databases were both searched using hmmsearch (−-cut_ga) [114] to predict carbohydrate-active enzymes (CAZymes) and pseudogenes respectively.

### Comparative pan-genomics

The new Sn15 genome reference was compared with draft assemblies of 18 *P. nodorum* isolates [20, 23] and 10 *P. avenae* isolates [BioProject: PRJNA476481], as well as long-read assemblies of 3 isolates of *P. nodorum*: Sn79–1087 [BioProject: PRJNA398070; Genome: GCA_002267025.1], Sn4 [BioProject: PRJNA398070; Genome: GCA_002267045.1] and Sn2000 [BioProject: PRJNA398070; Genome: GCA_002267005.1] [15, 31] (Supplementary Table 1). Whole-genome alignments and variant calling was performed via MUMmer v3 (nucmer --maxmatch, show-coords -T -H -r) [88] and the percent coverage of matches of each isolate relative to Sn15 was calculated within 50 Kbp windows via BEDtools v2.26.0 (makewindows, coverage) [87]. PAVs were calculated from nucmer (delta-filter − 1) alignments via BEDtools v2.26.0 (genomecov -bga) [87]. SNPs were calculated from nucmer alignments (show-snps -rlHTC) [88] and SNP density was calculated in 10 Kbp windows via BEDtools v2.26.0 (genomecov -bga) [87]. SNPs were analysed with SnpEff [115] relative to the new Sn15 gene annotations and the non-synonymous/synonymous mutation (Dn/Ds) ratio was calculated for every Sn15 gene both: 1) versus individual isolates and 2) averaged over all isolate comparisons. For visualisation by CIRCOS v 0.69–3 [116], Dn/Ds ratios were averaged across all genes within 50 Kbp windows via BEDtools v2.26.0 (map -c 4 -o mean) [87].

### Prediction of necrotrophic effector (NE) candidate gene loci

Putative effector genes were predicted based on the ranking of cumulative scores assigned from effector-associated gene or protein properties, as has been reported in previous studies [20, 23]. In this study the features (Supplementary Table 4) used to assign scores were: predicted secretion by SignalP 4.1 [111] (1 point); molecular weight < 30 kDa (1 point); %cysteines > 4% (1 point); DN:DS > 1.5 (1 point); distance of 0–5 from an AT-rich region as predicted by OcculterCut [26] (1 point); EffectorP [47] score > 0.9 (1 point); an *in planta* to in vitro differential expression ratio (IP:IV) > 5 (2 points) or < 1 (− 2 points); sub-telomeric location within genome (within 500 Kbp of sequence end, 2 points); presence (− 3 points) or absence (3 points) of ortholog in low-virulence isolate Sn79–1087; presence of ortholog predicted as an effector candidate in US isolate Sn4 [31] (3 points); assigned an effector/toxin-like functional annotation (3 points) and; predicted lateral-gene transfer event with a fungal pathogen species [79] (3 points).

## Supplementary Information

**Additional file 1: Supplementary Figure 1**. Comparison of non-repetitive regions of accessory chromosome 23 (AC23, red) to other Sn15 chromosomes (black), indicating that it is not the product of duplication of a core, sister chromosome. The GC content of AC23 is indicated by the linear plot, and local repeat density is indicated in the heat map below (red). Nucleotide matches > 200 bp are indicated by grey arcs

**Additional file 2: Supplementary Figure 2.** Comparison of non-repetitive regions of accessory chromosome 23 (AC23, black) of > 100 bp in length (grey arcs) to *P. tritici-repentis* BFP chromosomes 1,3 4, and 11 (red), *P. tritici-repentis* M4 chromosomes 1, 3, 4, 6 and 10 (green), *Bipolaris maydis* scaffold 16 (blue) and *B. sorokiniana* chromosomes 2, 4, and 9. This comparison indicated a trend of telomeric proximity in the relative matching regions of related species.

**Additional file 3: Supplementary Figure 3**. Alignment of locally collinear blocks (LCBs) via Mauve, indicating large sections of similarity with structural rearrangements between Chromosome 4 of *P. nodorum* isolate Sn15 (top) and corresponding chromosomal sequences of isolates Sn4, Sn2000, and Sn79–1087, presented at the whole chromosome level (A) and within ~ 700–800 Kb of the telomere (B).

**Additional file 4: Supplementary Table 1**. Summary of draft (A) and high-quality (B) genome assemblies of *Parastagonospora* spp. alternate isolates used in this study for comparative genomics versus the Australian reference isolate Sn15.

**Additional file 5: Supplementary Table 2**. Comparison of repetitive sequence masking in the new *P. nodorum* Sn15 genome assembly using 3 different repeat libraries applied sequentially in 3 iterations.

**Additional file 6: Supplementary Table 3.** De novo repeat sequences predicted within the *Parastagonospora nodorum* Sn15 genome assembly.

**Additional file 7: Supplementary Table 4**. Summary of *Parastagonospora nodorum* gene properties and predicted effector candidate genes.

**Additional file 8: Supplementary Table 5.** Summary of assembled sequence lengths in the new *P. nodorum* Sn15 genome assembly, and estimates of their potential to be unresolved by PFGE comparing a 1% size error range to the size difference with the next longest sequence.

**Additional file 9: Supplementary Table 6**. Properties of selected large regions of the Sn15 assembly exhibiting presence absence variation (PAV) across the *Parastagonospora* population.

**Additional file 10: Supplementary Table 7**. Presence-absence variation (PAV) matrix for comparison of *Parastagonospora nodorum* Sn15 genes versus all other *Parastagonospora* spp. isolates included in this study.

**Additional file 11: Supplementary Table 8**. Summary of scaffold sequences from Syme et al. 2013 corresponding to chromosomes of new optical map-assisted long-read genome assembly for *Parastagonospora nodorum* Sn15

**Additional file 12: Supplementary Table 9**. Summary of genes and their functional annotations within the Chromosome 8 PAV region.

**Additional file 13: Supplementary Table 10**. Summary of average SNP density and DN/DS selection metrics across the *Parastagonospora* spp. population, relative to the *P. nodorum* Sn15 reference genome assembly. )

**Additional file 14: Supplementary Table 11**. Summary of gene and effector candidate gene distances from nearest AT-rich regions in the *P. nodorum* Sn15 assembly.

**Additional file 15: Supplementary Table 12**. Orthology comparison between gene predictions of *P. nodorum* Sn15 and Sn4, indicating Sn15 orthologs to Sn4 effector candidates under diversifying selection identified in Richards et al. 2019.

**Additional file 16: Supplementary Table 13**. Summary of gene content and functional annotation for *P. nodorum* Sn15 accessory chromosome 23 (AC23).

**Additional file 17: Supplementary Text 1**. Notes on the *P. nodorum* Sn15 genome assembly and the integration of optical mapping data.

## Data Availability

The SN15 reference genome and protein data is available under BioProject: PRJNA686477. The updated SN15 genome assembly sequence consisting of 23 chromosomes is deposited under [Genome: GCA_016801405.1/ASM1680140v1; NUC: CP069023.1 - CP069045.1]. P. nodorum SN15 transcriptome data was previously deposited under BioProject:PRJNA632579. Alternate isolate data for *P. nodorum* and *P. avenae* was previously deposited under BioProject:PRJNA47648. Alternate reference isolate data was obtained for Sn79–1087 from BioProject:PRJNA398070 / GCA_002267025.1, for Sn4 from BioProject:PRJNA398070 / GCA_002267045.1, and for Sn2000 from BioProject:PRJNA398070 / GCA_002267005.1.

## Abbreviations

AC: Accessory chromosome
AR: Accessory region
BFB: Breakage-fusion bridge
EDTA: Ethylenediaminetetraacetic acid
IP: *In planta*
IV: In vitro
P5-C3: 5th generation polymerase, 3rd generation chemistry
PAV: Presence-absence variation
PFGE: Pulsed-field gel eletrophoresis
RIP: Repeat-induced point mutation
SMRT: Single-molecule real-time
SNB: Septoria nodorum blotch
SNP: Single nucleotide polymorphism
