# Supplementary material for: Chromosome-level genome assembly and manually-curated proteome of model necrotroph Parastagonospora nodorum Sn15 reveals a genome-wide trove of candidate effector homologs, and redundancy of virulence-related functions within an accessory chromosome
Source: BMC Genomics. 2021 May 25;22:382. doi: 10.1186/s12864-021-07699-8 (PMC8146201; doi:10.1186/s12864-021-07699-8)
Supplement: Supplementary file 1 — Additional file 1: Supplementary Figure 1. Comparison of non-repetitive regions of accessory chromosome 23 (AC23, red) to other Sn15 chromosomes (black), indicating that it is not the product of duplication of a core, sister chromosome. The GC content of AC23 is indicated by the linear plot, and local repeat density is indicated in the heat map below (red). Nucleotide matches > 200 bp are indicated by grey arcs [file 12864_2021_7699_MOESM1_ESM.docx]

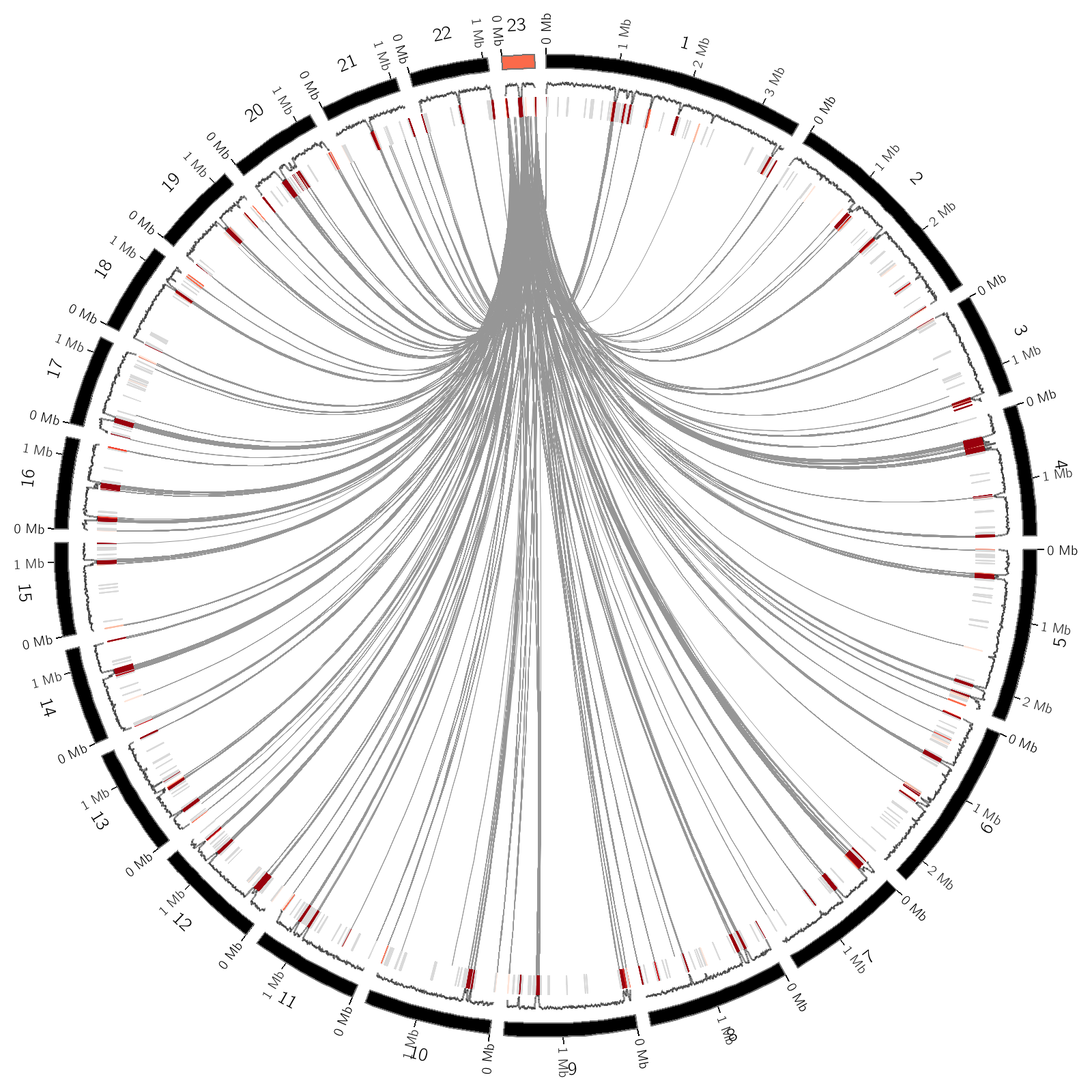


Supplementary Figure 1 **Comparison of non-repetitive regions of accessory chromosome 23 (AC23, red) to other Sn15 chromosomes (black), indicating that it is not the product of duplication of a core, sister chromosome. The GC content of AC23 is indicated by the linear plot, and local repeat density is indicated in the heat map below (red). Nucleotide matches >200 bp are indicated by grey arcs.**
