# Supplementary material for: Chromosome-level genome assembly and manually-curated proteome of model necrotroph Parastagonospora nodorum Sn15 reveals a genome-wide trove of candidate effector homologs, and redundancy of virulence-related functions within an accessory chromosome
Source: BMC Genomics. 2021 May 25;22:382. doi: 10.1186/s12864-021-07699-8 (PMC8146201; doi:10.1186/s12864-021-07699-8)
Supplement: Supplementary file 2 — Additional file 2: Supplementary Figure 2. Comparison of non-repetitive regions of accessory chromosome 23 (AC23, black) of > 100 bp in length (grey arcs) to P. tritici-repentis BFP chromosomes 1,3 4, and 11 (red), P. tritici-repentis M4 chromosomes 1, 3, 4, 6 and 10 (green), Bipolaris maydis scaffold 16 (blue) and B. sorokiniana chromosomes 2, 4, and 9. This comparison indicated a trend of telomeric proximity in the relative matching regions of related species. [file 12864_2021_7699_MOESM2_ESM.docx]

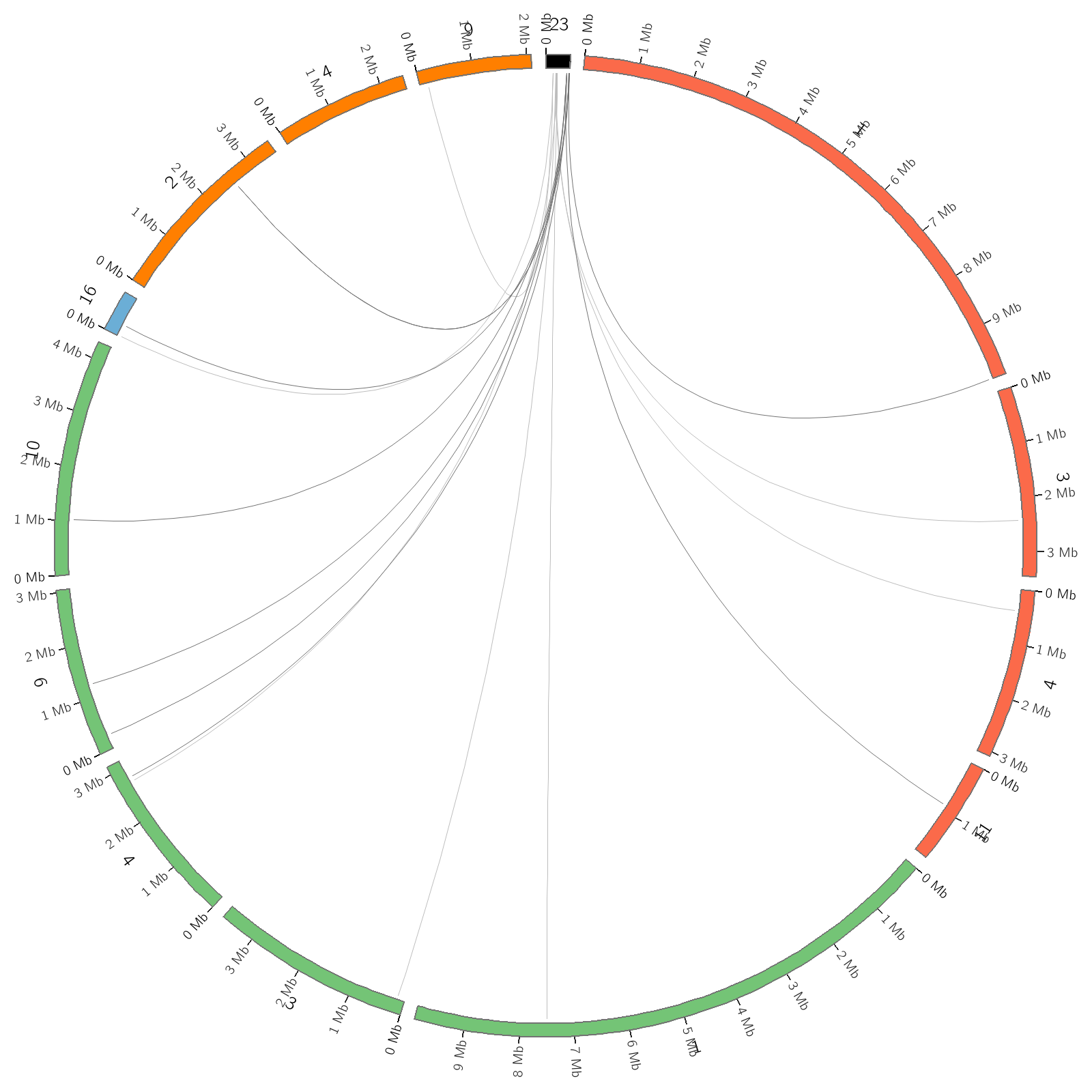


Supplementary Figure 2 Comparison of non-repetitive regions of accessory chromosome 23 (AC23, black) of >100 bp in length (grey arcs) to *P. tritici-repentis* BFP chromosomes 1,3 4, and 11 (red), *P. tritici-repentis* M4 chromosomes 1, 3, 4, 6 and 10 (green), *Bipolaris maydis* scaffold 16 (blue) and *B. sorokiniana* chromosomes 2, 4, and 9. This comparison indicated a trend of telomeric proximity in the relative matching regions of related species.
