# Supplementary material for: Chromosome-level genome assembly and manually-curated proteome of model necrotroph Parastagonospora nodorum Sn15 reveals a genome-wide trove of candidate effector homologs, and redundancy of virulence-related functions within an accessory chromosome
Source: BMC Genomics. 2021 May 25;22:382. doi: 10.1186/s12864-021-07699-8 (PMC8146201; doi:10.1186/s12864-021-07699-8)
Supplement: Supplementary file 3 — Additional file 3: Supplementary Figure 3. Alignment of locally collinear blocks (LCBs) via Mauve, indicating large sections of similarity with structural rearrangements between Chromosome 4 of P. nodorum isolate Sn15 (top) and corresponding chromosomal sequences of isolates Sn4, Sn2000, and Sn79–1087, presented at the whole chromosome level (A) and within ~ 700–800 Kb of the telomere (B). [file 12864_2021_7699_MOESM3_ESM.docx]

*
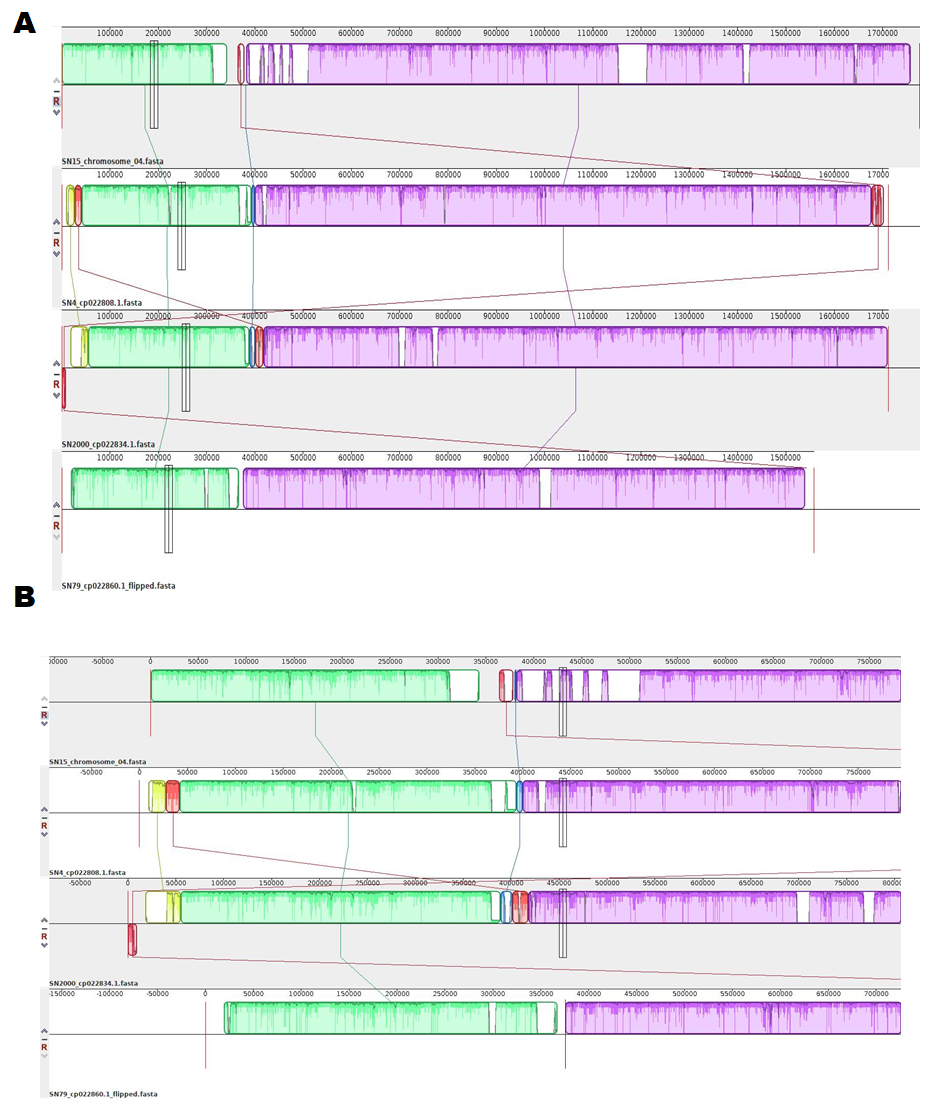
*

Supplementary Figure 3 Alignment of locally collinear blocks (LCBs) via Mauve, indicating large sections of similarity with structural rearrangements between Chromosome 4 of *P. nodorum* isolate Sn15 (top) and corresponding chromosomal sequences of isolates Sn4, Sn2000, and Sn79-1087, presented at the whole chromosome level (A) and within ~700-800 Kb of the telomere (B).
