# Supplementary material for: Chromosome-level genome assembly and manually-curated proteome of model necrotroph Parastagonospora nodorum Sn15 reveals a genome-wide trove of candidate effector homologs, and redundancy of virulence-related functions within an accessory chromosome
Source: BMC Genomics. 2021 May 25;22:382. doi: 10.1186/s12864-021-07699-8 (PMC8146201; doi:10.1186/s12864-021-07699-8)
Supplement: Supplementary file 4 — Additional file 4: Supplementary Table 1. Summary of draft (A) and high-quality (B) genome assemblies of Parastagonospora spp. alternate isolates used in this study for comparative genomics versus the Australian reference isolate Sn15. [file 12864_2021_7699_MOESM4_ESM.docx]

Supplementary Table 1 Summary of draft (A) and high-quality (B) genome assemblies of *Parastagonospora* spp. alternate isolates used in this study for comparative genomics versus the Australian reference isolate Sn15.

| **A) Species** | **Isolate** | **Location** | **Assembly length (Mbp)** | **Scaffolds** | **Genes** |
| --- | --- | --- | --- | --- | --- |
| *P. nodorum* | B2.1b | Iran | 37.38 | 2,906 | 14,765 |
| *P. nodorum* | C1.2a | Iran | 37.43 | 1,557 | 14,362 |
| *P. nodorum* | IR10_9.1a | Iran | 37.28 | 3,673 | 15,094 |
| *P. nodorum* | FIN-2 | Finland | 38.41 | 1,381 | 14,439 |
| *P. nodorum* | SWE-3 | Sweden | 37.85 | 1,714 | 14,676 |
| *P. nodorum* | Sn Cp2052 | Sweden | 37.32 | 3,026 | 14,953 |
| *P. nodorum* | BRSn9870 | Brazil | 41.23 | 4,911 | 15,930 |
| *P. nodorum* | Sn99CH 1A7a | Switzerland | 37.9 | 853 | 14,175 |
| *P. nodorum* | SnChi01 40a | China | 37.88 | 779 | 14,208 |
| *P. nodorum* | SnSA95.103 | South Africa | 49.94 | 11,772 | 17,034 |
| *P. nodorum* | AR1-1 | Arkansas, USA | 36.61 | 882 | 14,217 |
| *P. nodorum* | GA9-1 | Georgia, USA | 36.53 | 664 | 14,201 |
| *P. nodorum* | MD4-1 | Maryland, USA | 36.53 | 701 | 14,216 |
| *P. nodorum* | VA 5-2 | Virginia, USA | 36.48 | 1,115 | 14,216 |
| *P. nodorum* | OH03 Sn-1501 | Ohio, USA | 37.1 | 1,281 | 14,297 |
| *P. nodorum* | SNOV92X D1.3 | Texas, USA | 36.66 | 785 | 14,220 |
| *P. nodorum* | SnOre11-1 | Oregon, USA | 37.42 | 748 | 14,158 |
| *P. nodorum* | WAC8410 | Australia | 40.27 | 384 | 14,768 |
| *P. avenae f.sp. triticea 1* | IR10_5.2b | Iran | 35.51 | 1,681 | 14,267 |
| *P. avenae f.sp. triticea 1* | Hartney99 | Canada | 36.58 | 3,381 | 16,342 |
| *P. avenae f.sp. triticea 1* | Jansen#4_55 | Canada | 32.06 | 10,109 | 16,342 |
| *P. avenae f.sp. triticea 5* | 82-4841 | North Dakota, USA | 38.53 | 2,444 | 14,167 |
| *P. avenae f.sp. triticea 5* | 83-6011-2 | North Dakota, USA | 37.52 | 2,367 | 14,127 |
| *P. avenae f.sp. triticea 4* | SN11IR_2_1.1 | Iran | 41.54 | 5,762 | 14,149 |
| *P. avenae f.sp. triticea 6* | SN11IR_6_1.1 | Iran | 33.51 | 1,174 | 12,290 |
| *P. avenae f.sp. triticea 6* | SN11IR_7_2.3 | Iran | 33.6 | 2,215 | 12,451 |
| *P. avenae f.sp. avenaria* | Mt.Barker | Washington, USA | 34.14 | 8,309 | 15,524 |
| *P. avenae f.sp. avenaria* | s258 | Netherlands | 39.49 | 4,090 | 13,984 |
| P2 group host: *T. aestivum* | A1 3.1a | Iran | 38.68 | 1,613 | 15,181 |
| P2 group host: *T. aestivum* | H6.2b | Iran | 39.05 | 1,764 | 15,052 |
| **B ) Species** | **Isolate** | **Location** | **Assembly length (Mbp)** | **Scaffolds** | **Genes** |
| P. nodorum | Sn4 | North Dakota, USA | 37,69 | 23 | 13454 |
| P. nodorum | Sn79-1087 | North Dakota, USA | 34,99 | 22 | 13253 |
| P. nodorum | Sn2000 | North Dakota, USA | 37,46 | 23 | n/a |
