# Supplementary material for: Chromosome-level genome assembly and manually-curated proteome of model necrotroph Parastagonospora nodorum Sn15 reveals a genome-wide trove of candidate effector homologs, and redundancy of virulence-related functions within an accessory chromosome
Source: BMC Genomics. 2021 May 25;22:382. doi: 10.1186/s12864-021-07699-8 (PMC8146201; doi:10.1186/s12864-021-07699-8)
Supplement: Supplementary file 5 — Additional file 5: Supplementary Table 2. Comparison of repetitive sequence masking in the new P. nodorum Sn15 genome assembly using 3 different repeat libraries applied sequentially in 3 iterations. [file 12864_2021_7699_MOESM5_ESM.docx]

Supplementary Table 2 Comparison of repetitive sequence masking in the new *P. nodorum* Sn15 genome assembly using 3 different repeat libraries applied sequentially in 3 iterations.

|  | **Iteration 1** | **Iteration 2** | **Iteration 3** | **Total** |
| --- | --- | --- | --- | --- |
| Repeat Consensus Sequence Library | Sn15 [1, 24, 25] | RepBase (taxon “Fungi”) [91] | *de novo* – RepeatModeler [93] | *na* |
| Repeats | 25 | 2505 | 14 | 2544 |
| Total Sequence  (Mbp / (%) | 2.59 Mbp  (6.85 %) | 0.1 Mbp  (0.27 %) | 0.24 Mbp  (0.63 %) | 2.93 Mbp |
