# Supplementary material for: Chromosome-level genome assembly and manually-curated proteome of model necrotroph Parastagonospora nodorum Sn15 reveals a genome-wide trove of candidate effector homologs, and redundancy of virulence-related functions within an accessory chromosome
Source: BMC Genomics. 2021 May 25;22:382. doi: 10.1186/s12864-021-07699-8 (PMC8146201; doi:10.1186/s12864-021-07699-8)
Supplement: Supplementary file 8 — Additional file 8: Supplementary Table 5. Summary of assembled sequence lengths in the new P. nodorum Sn15 genome assembly, and estimates of their potential to be unresolved by PFGE comparing a 1% size error range to the size difference with the next longest sequence. [file 12864_2021_7699_MOESM8_ESM.docx]

Supplementary Table 5 **Summary of assembled sequence lengths in the new *P. nodorum* Sn15 genome assembly, and estimates of their potential to be unresolved by PFGE comparing a 1% size error range to the size difference with the next longest sequence.**

| **Chr** | **Length (bp)** | **Size difference (bp)** | **(1% error range)** | **Potentially unresolvable?** |
| --- | --- | --- | --- | --- |
| **1** | **3,547,281** | **-** |  |  |
| **2** | **2,905,030** | **642,251** | **29053** | **no** |
| **5** | **2,339,425** | **565,605** | **23395** | **no** |
| **6** | **2,332,126** | **7,299** | **23326** | **yes** |
| **8** | **1,927,844** | **404,282** | **19274** | **no** |
| **9** | **1,800,813** | **127,031** | **18003** | **no** |
| **4** | **1,777,463** | **23,350** | **17773** | **no** |
| **10** | **1,722,205** | **55,258** | **17225** | **no** |
| **7** | **1,683,166** | **39,039** | **16836** | **no** |
| **11** | **1,470,553** | **212,613** | **14703** | **no** |
| **12** | **1,457,434** | **13,119** | **14574** | **yes** |
| **13** | **1,455,851** | **1,583** | **14551** | **yes** |
| **3** | **1,391,116** | **64,735** | **13916** | **no** |
| **14** | **1,308,001** | **83,115** | **13081** | **no** |
| **15** | **1,262,249** | **45,752** | **12629** | **no** |
| **16** | **1,240,242** | **22,007** | **12402** | **no** |
| **17** | **1,222,753** | **17,489** | **12223** | **no** |
| **18** | **1,204,143** | **18,610** | **12043** | **no** |
| **20** | **1,202,167** | **1,976** | **12027** | **yes** |
| **19** | **1,190,348** | **11,819** | **11908** | **yes** |
| **21** | **1,067,918** | **122,430** | **10678** | **no** |
| **22** | **1,067,756** | **162** | **10676** | **yes** |
| **23** | **444,753** | **623,003** | **4443** | **no** |
