# Supplementary material for: Chromosome-level genome assembly and manually-curated proteome of model necrotroph Parastagonospora nodorum Sn15 reveals a genome-wide trove of candidate effector homologs, and redundancy of virulence-related functions within an accessory chromosome
Source: BMC Genomics. 2021 May 25;22:382. doi: 10.1186/s12864-021-07699-8 (PMC8146201; doi:10.1186/s12864-021-07699-8)
Supplement: Supplementary file 9 — Additional file 9: Supplementary Table 6. Properties of selected large regions of the Sn15 assembly exhibiting presence absence variation (PAV) across the Parastagonospora population. [file 12864_2021_7699_MOESM9_ESM.docx]

Supplementary Table 6 Properties of selected large regions of the Sn15 assembly exhibiting presence absence variation (PAV) across the *Parastagonospora* population.

|  | **Whole genome** | **AC23** | **Chr 4 ToxA region** | **Chr7 Duplication** | **Chr 10 (scaffold 69)** | **CH8 (scaffold 50)** |
| --- | --- | --- | --- | --- | --- | --- |
| **Type** | n/a | Accessory chromosome [20] | Laterally-transferred, repeat-rich, effector region [20] | Potentially duplicated region | Potential accessory region [23] | putative accessory region [23, 29] |
| **Location** |  | Chr 23: 1-444,753 | Chr 4: 368,229-442,545 | Chr 7: 154,291 – 609,449 | Chr 10: 352,515 – 362,116 | Chr 8: 340,499 – 428,423 |
| **Length (bp)** |  | 444,753 | 74,316 | 455,158 | 9,610 | 87,924 |
| **GC content (%)** | 50.2 | 48.1 | 31.9 | 50.2 | 36.7 | 46.6 |
| **Repeat content (%)** | 8.2 | 14.8 | 59.8 | 5.7 | 99.9 | 13.9 |
| **SNP coverage (%)** | 2.4 | 1.1 | 0.7 | 2.5 | na | 2.2 |
| **Genes (SetA)** | 13893 | 144 | 9 | 169 | 0 | 27 |
| **Avg. DN/DS** | 0.7 \| 0.9 | 1.9 \| 1.9 | 1.3 \| 1.3 | 0.6 \| 1.0 | na \| 1.8 | 0.5 \| 0.9 |
| **Functionally annotated genes** | 9505 | 27 | 4 \| 5 | 114 \| 114 | 0 \| 1 | 19 \| 19 |
| **Secreted genes** | 1588 | 7 | 1 | 15 | 0 | 6 |
| **Candidate effector genes** | 257 | 4 | 1 | 4 | 0 | 0 |

Supplementary Table 7 **Presence-absence variation (PAV) matrix for comparison of *Parastagonospora nodorum* Sn15 genes versus all other *Parastagonospora* spp. isolates included in this study**
