# Supplementary material for: Chromosome-level genome assembly and manually-curated proteome of model necrotroph Parastagonospora nodorum Sn15 reveals a genome-wide trove of candidate effector homologs, and redundancy of virulence-related functions within an accessory chromosome
Source: BMC Genomics. 2021 May 25;22:382. doi: 10.1186/s12864-021-07699-8 (PMC8146201; doi:10.1186/s12864-021-07699-8)
Supplement: Supplementary file 11 — Additional file 11: Supplementary Table 8. Summary of scaffold sequences from Syme et al. 2013 corresponding to chromosomes of new optical map-assisted long-read genome assembly for Parastagonospora nodorum Sn15 [file 12864_2021_7699_MOESM11_ESM.docx]

Supplementary Table 8 **Summary of scaffold sequences from Syme et al. 2013 corresponding to chromosomes of new optical map-assisted long-read genome assembly for *Parastagonospora nodorum* Sn15**

| Chromosome | Optical map estimated physical length (bp) | Assembly length (bp, % of 39.2 Mbp) | 5’ and 3’ telomeric repeat | Scaffold (Syme et al. 2013/2018) | Notes |  |
| --- | --- | --- | --- | --- | --- | --- |
|  |  |  |  |  |  |  |
|  |  |  |  |  |  |  |
| 1 | 3549748 | 3547281  (99.9%) | yes/yes | scaffold_015 |  |  |
|  |  |  |  | scaffold_001 |  |  |
| 2 | 2908472 | 2905030 (99.9%) | yes/yes | scaffold_003 |  |  |
|  |  |  |  | scaffold_012 |  |  |
| 3 | 2423570 | 1391116 (57.4%) | yes/no | scaffold_005 | ~1 Mbp of rDNA repeat (NOR) |  |
|  |  |  |  |  |  |  |
| 4 | 2395026 | 1777463 (74.2%) | yes/yes | scaffold_039 | large  (~570 Kb) region TE-rich |  |
|  |  |  |  | scaffold_051 |  |  |
|  |  |  |  | scaffold_011 |  |  |
| 5 | 2347804 | 2339425 (99.6%) | yes/yes | scaffold_034 |  |  |
|  |  |  |  | scaffold_004 |  |  |
|  |  |  |  | scaffold_041 |  |  |
| 6 | 2335482 | 2332126 (99.8%) | yes/yes | scaffold_008 |  |  |
|  |  |  |  | scaffold_002 |  |  |
|  |  |  |  |  |  |  |
| 7 | 2192988 | 1683166 (76.7%) | yes/yes | scaffold_020 | ~460 Kb sequence potential inverted duplication |  |
|  |  |  |  | scaffold_020 |  |  |
|  |  |  |  | scaffold_013 |  |  |
| 8 | 1942711 | 1927844 (99.2%) | no/yes | scaffold_017 |  |  |
|  |  |  |  | scaffold_050 |  |  |
|  |  |  |  | scaffold_006 |  |  |
| 9 | 1810910 | 1800813 (99.4%) | yes/yes | scaffold_009 |  |  |
|  |  |  |  | scaffold_031 |  |  |
| 10 | 1714514 | 1722205 (100.4%) | yes/yes | scaffold_035 |  |  |
|  |  |  |  | scaffold_010 |  |  |
| 11 | 1488386 | 1470553 (98.8%) | yes/yes | scaffold_014 |  |  |
|  |  |  |  | scaffold_037 |  |  |
| 12 | 1474181 | 1457434 (98.9%) | yes/yes | scaffold_040 |  |  |
|  |  |  |  | scaffold_021 |  |  |
|  |  |  |  | scaffold_032 |  |  |
| 13 | 1461681 | 1455851 (99.6%) | yes/yes | scaffold_007 |  |  |
|  |  |  |  | scaffold_082 |  |  |
|  |  |  |  | scaffold_007 |  |  |
| 14 | 1311505 | 1308001 (99.7%) | yes/yes | scaffold_019 |  |  |
|  |  |  |  | scaffold_030 |  |  |
| 15 | 1260933 | 1262249 (100.1%) | yes/yes | scaffold_016 |  |  |
|  |  |  |  | scaffold_042 |  |  |
| 16 | 1249355 | 1240242 (99.3%) | yes/yes | scaffold_047 |  |  |
|  |  |  |  | scaffold_028 |  |  |
|  |  |  |  | scaffold_099 |  |  |
|  |  |  |  | scaffold_028 |  |  |
|  |  |  |  | scaffold_024 |  |  |
| 17 | 1226944 | 1222753 (99.7%) | yes/yes | scaffold_043 |  |  |
|  |  |  |  | scaffold_017 |  |  |
| 18 | 1205117 | 1204143 (99.9%) | yes/yes | scaffold_018 |  |  |
|  |  |  |  | scaffold_038 |  |  |
| 19 | 1191245 | 1190348 (99.9%) | yes/yes | scaffold_008 |  |  |
| 20 | 1193615 | 1202167 (100.7%) | yes/yes | scaffold_033 |  |  |
|  |  |  |  | scaffold_020 |  |  |
| 21 | 1066034 | 1067918 (100.2%) | yes/yes | scaffold_027 |  |  |
|  |  |  |  | scaffold_023 |  |  |
| 22 | 1069038 | 1067756 (99.9%) | yes/yes | scaffold_022 |  |  |
|  |  |  |  | scaffold_025 |  |  |
| 23 | 443105 | 444753 (100.4%) | yes/yes | scaffold_045 |  |  |
|  |  |  |  | scaffold_044 |  |  |
| Unplaced | NA | 317211 (0.8%) | NA |  |  |  |
