# Supplementary material for: Chromosome-level genome assembly and manually-curated proteome of model necrotroph Parastagonospora nodorum Sn15 reveals a genome-wide trove of candidate effector homologs, and redundancy of virulence-related functions within an accessory chromosome
Source: BMC Genomics. 2021 May 25;22:382. doi: 10.1186/s12864-021-07699-8 (PMC8146201; doi:10.1186/s12864-021-07699-8)
Supplement: Supplementary file 12 — Additional file 12: Supplementary Table 9. Summary of genes and their functional annotations within the Chromosome 8 PAV region. [file 12864_2021_7699_MOESM12_ESM.docx]

Supplementary Table 9 Summary of genes and their functional annotations within the Chromosome 8 PAV region.

| **Locus ID** | **start** | **end** | **strand** | **length** | **annotation set** | **secreted** | **function** |
| --- | --- | --- | --- | --- | --- | --- | --- |
| SNOR_16523 | 350448 | 351487 | - | 1039 | A | Y | Metallopeptidase |
| SNOR_16524 | 352868 | 353734 | + | 866 | A |  |  |
| SNOR_16525 | 358789 | 359957 | + | 1168 | A |  | Fe(2+) 2-oxoglutarate dioxygenase, Oxoglutarate/iron-dependent dioxygenase, Isopenicillin N synthase-like |
| SNOR_16526 | 360466 | 361633 | + | 1167 | A |  | Pirin, RmlC-like cupin domain |
| SNOR_16527 | 361733 | 362787 | - | 1054 | A | Y |  |
| SNOR_16528 | 363302 | 365202 | - | 1900 | A |  |  |
| SNOR_16529 | 366419 | 369850 | - | 3431 | A |  |  |
| SNOR_30986 | 370893 | 373178 | - | 2285 | A |  | RNA exonuclease 1-like/REX1-like RNase T/DNA polymerase III\| Ribonuclease H-like superfamily |
| SNOR_16531 | 373423 | 374562 | - | 1139 | A |  | N-acyl-phosphatidylethanolamine-hydrolysing phospholipase D, Metallo-hydrolase/oxidoreductase superfamily |
| SNOR_16533 | 375190 | 376532 | + | 1342 | A | Y | Ribonuclease T2 |
| SNOR_16534 | 376832 | 378807 | - | 1975 | A |  | Tetratricopeptide repeat (TPR) domain, Mitochondrial outer membrane translocase complex, Tom20 domain superfamily |
| SNOR_16535 | 379357 | 380421 | - | 1064 | A |  | Eukaryotic RNA Recognition Motif (RRM) profile. RNA recognition motif domain |
| SNOR_16537 | 380869 | 384284 | + | 3415 | A |  | Adenosine/AMP deaminase, Metal-dependent hydrolase |
| SNOR_16538 | 384485 | 385188 | - | 703 | A |  |  |
| SNOR_16539 | 385721 | 387977 | + | 2256 | A |  | Armadillo-like helical\|ELMO/CED-12 domain , Domain of unknown function (DUF3361, DUF3361), Pleckstrin-like, PH-like domain |
| SNOR_16541 | 389274 | 390963 | + | 1689 | A |  |  |
| SNOR_16543 | 393917 | 395720 | - | 1803 | A |  | Tailless complex polypeptide 1 (TCP-1)/cpn60 chaperonin family Chaperonin, GroEL-like domain superfamily\| GroEL-like equatorial domain superfamily\| GroEL-like apical domain |
| SNOR_16544 | 396046 | 397472 | + | 1426 | A |  | AAA+ ATPase domain, 26Sp45: 26S proteasome subunit P45 |
| SNOR_16545 | 397652 | 398437 | + | 785 | A |  |  |
| SNOR_16547 | 398762 | 401018 | - | 2256 | A |  | FAD binding domain FAD-dependent oxidoreductase 2, Succinate dehydrogenase/fumarate reductase flavoprotein |
| SNOR_16548 | 402683 | 404014 | - | 1331 | A |  | Ribosomal protein L3 |
| SNOR_16549 | 404560 | 406342 | + | 1782 | A |  | WD40-repeat-containing domain, Protein phosphatase 2A regulatory subunit PR55 (55kDa regulatory subunit) |
| SNOR_16550 | 407327 | 408526 | + | 1199 | A | Y | UAA family UDP-galactose transporter |
| SNOR_30987 | 408976 | 411397 | + | 2421 | A |  | MIR domain, protein-O-mannosyltransferase, Dolichyl-phosphate-mannose-protein mannosyltransferase Glycosyl transferase family 39/83 |
| SNOR_16552 | 412572 | 417109 | + | 4537 | A | Y | Sortilin, neurotensin receptor 3, WD40/YVTN repeat-like-containing domain superfamily, Sialidase (non-viral) |
| SNOR_16554 | 417951 | 418724 | - | 773 | A |  |  |
| SNOR_16555 | 418843 | 421056 | - | 2213 | A | Y | Subtilisin/Subtilase, Peptidase S8/S53 domain, Proteinase-K-like, Serine protease |
