# Supplementary material for: Chromosome-level genome assembly and manually-curated proteome of model necrotroph Parastagonospora nodorum Sn15 reveals a genome-wide trove of candidate effector homologs, and redundancy of virulence-related functions within an accessory chromosome
Source: BMC Genomics. 2021 May 25;22:382. doi: 10.1186/s12864-021-07699-8 (PMC8146201; doi:10.1186/s12864-021-07699-8)
Supplement: Supplementary file 13 — Additional file 13: Supplementary Table 10. Summary of average SNP density and DN/DS selection metrics across the Parastagonospora spp. population, relative to the P. nodorum Sn15 reference genome assembly. ) [file 12864_2021_7699_MOESM13_ESM.docx]

Supplementary Table 10 **Summary of average SNP density and DN/DS selection metrics across the *Parastagonospora* spp. population, relative to the *P. nodorum* Sn15 reference genome assembly.**

| CHROMOSOME | SNP coverage average across all isolates | SNP coverage delta from avg | DNDS average aross all isolates | DNDS delta from avg |
| --- | --- | --- | --- | --- |
| 1 | 0.025392 | 0.00118 | 0.760 | -0.163 |
| 2 | 0.025390 | 0.00118 | 0.903 | -0.021 |
| 3 | 0.025472 | 0.00126 | 0.968 | 0.044 |
| 4 | 0.023343 | -0.00087 | 1.038 | 0.114 |
| 5 | 0.024681 | 0.00047 | 1.004 | 0.081 |
| 6 | 0.024735 | 0.00052 | 1.031 | 0.107 |
| 7 | 0.024395 | 0.00018 | 0.887 | -0.037 |
| 8 | 0.024833 | 0.00062 | 0.943 | 0.019 |
| 9 | 0.023926 | -0.00029 | 0.856 | -0.068 |
| 10 | 0.025171 | 0.00096 | 0.944 | 0.020 |
| 11 | 0.024817 | 0.00060 | 0.962 | 0.038 |
| 12 | 0.023802 | -0.00041 | 0.812 | -0.112 |
| 13 | 0.024583 | 0.00037 | 0.920 | -0.004 |
| 14 | 0.023874 | -0.00034 | 0.919 | -0.004 |
| 15 | 0.023541 | -0.00067 | 0.907 | -0.017 |
| 16 | 0.023360 | -0.00085 | 0.833 | -0.091 |
| 17 | 0.022509 | -0.00170 | 0.971 | 0.047 |
| 18 | 0.024979 | 0.00077 | 0.969 | 0.045 |
| 19 | 0.023858 | -0.00035 | 0.919 | -0.004 |
| 20 | 0.021575 | -0.00264 | 0.836 | -0.088 |
| 21 | 0.024346 | 0.00013 | 0.815 | -0.109 |
| 22 | 0.023332 | -0.00088 | 0.906 | -0.018 |
| 23 | 0.011564 | -0.01265 | 1.912 | 0.988 |
| Whole genome | 0.024212 |  | 0.924 |  |
|  | min | -0.01265 | min | -0.163 |
|  | max | 0.00126 | max | 0.988 |
