# Supplementary material for: Chromosome-level genome assembly and manually-curated proteome of model necrotroph Parastagonospora nodorum Sn15 reveals a genome-wide trove of candidate effector homologs, and redundancy of virulence-related functions within an accessory chromosome
Source: BMC Genomics. 2021 May 25;22:382. doi: 10.1186/s12864-021-07699-8 (PMC8146201; doi:10.1186/s12864-021-07699-8)
Supplement: Supplementary file 14 — Additional file 14: Supplementary Table 11. Summary of gene and effector candidate gene distances from nearest AT-rich regions in the P. nodorum Sn15 assembly. [file 12864_2021_7699_MOESM14_ESM.docx]

Supplementary Table 11 **Summary of gene and effector candidate gene distances from nearest AT-rich regions in the *P. nodorum* Sn15 assembly.**

| Distance from nearest AT-rich region (nt) | Set A transcripts number (% of SetA total) | Effector candidates (% of total genes) |
| --- | --- | --- |
|  |  |  |
| 0-500 | 110 (0.78%) | 4 (0.997%) |
| 500-1000 | 49 (0.35%) | 5 (1.246%) |
| 0-5k | 345 (2.44%) | 26 (6.484%) |
| 5k-10k | 403 (2.85%) | 13 (3.241%) |
| 10k-15k | 403 (2.845%) | 10 (2.493%) |
| 15k-20k | 369 (2.605%) | 14 (3.491%) |
| 20k-25k | 413 (2.915%) | 19 (4.738%) |
| 25k-30k | 370 (2.612%) | 8 (1.995%) |
| 30k-35k | 364 (2.569%) | 10 (2.493%) |
| 35k-40k | 339 (2.393%) | 10 (2.493%) |
| 40k-45k | 362 (2.555%) | 7 (1.745%) |
| 45k-50k | 324 (2.287%) | 11 (2.743%) |
| >50000 | 10,313 (72.81%) | 273 (68.079%) |
