# Supplementary material for: Chromosome-level genome assembly and manually-curated proteome of model necrotroph Parastagonospora nodorum Sn15 reveals a genome-wide trove of candidate effector homologs, and redundancy of virulence-related functions within an accessory chromosome
Source: BMC Genomics. 2021 May 25;22:382. doi: 10.1186/s12864-021-07699-8 (PMC8146201; doi:10.1186/s12864-021-07699-8)
Supplement: Supplementary file 16 — Additional file 16: Supplementary Table 13. Summary of gene content and functional annotation for P. nodorum Sn15 accessory chromosome 23 (AC23). [file 12864_2021_7699_MOESM16_ESM.docx]

Supplementary Table 13 Summary of gene content and functional annotation for *P. nodorum* Sn15 accessory chromosome 23 (AC23).

| **Locus ID** | **isoform** | **annotation set** | **Start** | **end** | **strand** | **length (bp)** | **secreted** | **effectorp** | **function** | **nearest AT-rich region (bp)** | **DN:DS** | **Cysteine residues** | **length (aa)** | **Molecular weight (kDa)** |
| --- | --- | --- | --- | --- | --- | --- | --- | --- | --- | --- | --- | --- | --- | --- |
| SNOR_16274 |  | A | 28675 | 30960 | + | 2285 |  |  | Ulp1 protease | 13482 | 1.18 | 4 | 761 | 84.6 |
| SNOR_16283 |  | A | 48421 | 49592 | + | 1171 |  |  | Prokaryotic membrane lipoprotein lipid attachment site | 33228 | 2.71 | NA | NA | NA |
| SNOR_16303 |  | A | 90913 | 92373 | + | 1460 |  |  | Leucine-rich repeat domain | 75803 | 0.95 | 9 | 441 | 49.8 |
| SNOR_16310 |  | A | 105992 | 107448 | + | 1456 |  |  | RING/FYVE/PHD-type Zinc finger | 90281 | 1.44 | NA | NA | NA |
| SNOR_42465 |  | B | 111753 | 112037 | - | 284 | Y | 0.87 |  | 85750 | 0.5 | 3 | 64 | 7.4 |
| SNOR_42468 |  | B | 115457 | 115720 | - | 263 | Y |  |  | 82006 | 1 | 5 | 86 | 9.1 |
| SNOR_16330 |  | A | 141063 | 141731 | - | 668 |  |  | DNA polymerase, palm domain | 55995 | 1.23 | 6 | 222 | 24.2 |
| SNOR_16333 |  | A | 148873 | 149708 | - | 835 |  |  | RING/FYVE/PHD-type Zinc finger | 48018 | 1.63 | 9 | 235 | 26 |
| SNOR_16353 |  | A | 182049 | 183538 | - | 1489 |  |  | Winged helix-like DNA-binding domain, S-adenosyl-L-methionine (SAM)-dependent O-methyltransferase class II | 14188 | 0.49 | 4 | 419 | 47.2 |
| SNOR_16354 |  | A | 183783 | 184786 | - | 1003 |  |  | S-adenosyl-L-methionine-dependent methyltransferase | 12940 | 0.29 | NA | NA | NA |
| SNOR_16355 |  | A | 185145 | 186659 | + | 1514 |  |  | Group II E-class P450 signature Cytochrome P450, CYP52 | 11070 | 0.55 | 4 | 504 | 57.4 |
| SNOR_16356 | A | A | 187377 | 188411 | + | 603 |  |  | Domain of unknown function (DUF3328) Mycotoxin biosynthesis protein UstYa-like | 9741 | 0.74 | 2 | 175 | 19.8 |
| SNOR_16356 | B | A | 187377 | 188411 | + | 1034 |  |  |  | 9386 | 0.74 | 6 | 258 | 29.5 |
| SNOR_16357 |  | A | 188504 | 189354 | - | 850 |  |  |  | 8372 | 0.73 | NA | NA | NA |
| SNOR_16358 |  | A | 190043 | 190524 | + | 481 | Y |  |  | 7260 | 0.49 | NA | NA | NA |
| SNOR_16360 |  | A | 191029 | 192070 | - | 1041 | Y |  | Alpha/beta hydrolase | 5656 | 4 | NA | NA | NA |
| SNOR_16362 |  | A | 194888 | 197410 | - | 2522 |  |  | Phenylalanine and histidine ammonia-lyases (PAL-HAL) aromatic amino acid lyase, L-Aspartase-like | 370 | 0.77 | 7 | 771 | 83.5 |
| SNOR_16268 |  | A | 249321 | 249934 | - | 613 |  |  | Valyl-tRNA synthetase | 6167 | 2.06 | 1 | 182 | 19.9 |
| SNOR_16259 |  | A | 264935 | 265852 | + | 712 |  |  | SET domain | 22055 | 0.64 | NA | NA | NA |
| SNOR_16241 |  | A | 293409 | 294254 | + | 845 |  |  | Insect cysteine-rich antifreeze protein | 2579 | 1 | NA | NA | NA |
| SNOR_16236 |  | A | 303594 | 304270 | - | 676 | Y | 1 |  | 4800 | 3.77 | 3 | 153 | 16.9 |
| SNOR_16226 |  | A | 321367 | 322573 | + | 691 | Y | 0.94 |  | 23085 | 2.75 | 2 | 179 | 19.9 |
| SNOR_16225 |  | A | 322964 | 325272 | + | 2308 |  |  | Proline rich extensin-like | 24169 | 2.11 | NA | NA | NA |
| SNOR_16223 |  | A | 326806 | 328388 | + | 1582 |  |  | Kinesin heavy chain motor domain, P-loop containing nucleoside triphosphate hydrolase | 28009 | 2.39 | 2 | 499 | 55.2 |
| SNOR_16214 |  | A | 342994 | 345471 | - | 2477 |  |  | Ulp1 protease | 44200 | 1.08 | 12 | 825 | 91.3 |
| SNOR_16213 |  | A | 346033 | 346908 | + | 875 |  |  | Aminoacyl-tRNA tRNA synthetase class I (I, L, M and V) | 47236 | 1.92 | 3 | 291 | 32.6 |
| SNOR_42478 |  | B | 346869 | 347216 | - | 347 |  |  | Nucleolar protein | 48109 | 1.75 | 1 | 89 | 10.5 |
| SNOR_16211 |  | A | 347373 | 348212 | + | 839 |  |  | Valyl-tRNA synthetase, Valine-tRNA ligase, Aminoacyl-tRNA synthetase class I (I, L, M and V) | 48576 | 1.3 | 6 | 279 | 31.4 |
| SNOR_16196 |  | A | 381787 | 382243 | - | 456 | Y |  |  | 51922 | 1.37 | 1 | 135 | 14.5 |
| SNOR_30965 |  | A | 386062 | 386892 | + | 830 |  |  | Reverse transcriptase | 47276 | 1.03 | 5 | 276 | 31.2 |
| SNOR_16178 |  | A | 411832 | 413217 | + | 1385 |  |  | Protein kinase | 20951 | 0.64 | 5 | 461 | 51.2 |
| SNOR_16175 |  | A | 416790 | 418802 | - | 2012 |  |  | GH18 Serine/threonine-protein kinase | 15363 | 1.26 | 11 | 581 | 68 |
| SNOR_16169 |  | A | 429448 | 431253 | - | 1805 |  |  | Trimeric LpxA-like superfamily, Maltose/galactoside / GAL4 Zn(2)-C6 fungal-type DNA-binding domain, Hexapeptide repeat | 2991 | 2.62 | 14 | 538 | 59.1 |
| SNOR_16168 |  | A | 432483 | 433850 | - | 1367 |  |  | MYND-type Zinc finger | 315 | 1.05 | 11 | 455 | 51.9 |
