# Supplementary material for: Chromosome-level genome assembly and manually-curated proteome of model necrotroph Parastagonospora nodorum Sn15 reveals a genome-wide trove of candidate effector homologs, and redundancy of virulence-related functions within an accessory chromosome
Source: BMC Genomics. 2021 May 25;22:382. doi: 10.1186/s12864-021-07699-8 (PMC8146201; doi:10.1186/s12864-021-07699-8)

# Supplementary Text

Supplementary Text 1 Notes on the *P. nodorum* Sn15 genome assembly and the integration of optical mapping data

**Assembly Completeness was assessed with BUSCO (v5.1.2) versus the “fungi” database (as of 14/3/2021)**

# The lineage dataset is: fungi_odb10 (Creation date: 2020-09-10, number of species: 549, number of BUSCOs: 758)

# BUSCO was run in mode: genome

# Gene predictor used: metaeuk

| Complete BUSCOs (C) | 751 | 99.1% |
| --- | --- | --- |
| Complete and single-copy BUSCOs (S) | 749 | 98.8% |
| Complete and duplicated BUSCOs (D) | 2 | 0.3% |
| Fragmented BUSCOs (F) | 3 | 0.4% |
| Missing BUSCOs (M) | 4 | 0.5% |
| Total BUSCO groups searched | 758 | 100.0% |

Summary of missing, duplicated and fragmented BUSCOs

| # Busco id | Status | Sequence | Gene Start | Gene End | Strand | Score | Length | OrthoDB url | Description |
| --- | --- | --- | --- | --- | --- | --- | --- | --- | --- |
| 20600at4751 | Duplicated | Chromosome_02 | 2203379 | 2211772 | + | 1300.5 | 785 | https://www.orthodb.org/v10?query=20600at4751 | glycine dehydrogenase |
| 20600at4751 | Duplicated | Chromosome_02 | 2208489 | 2211772 | + | 1306.2 | 785 | https://www.orthodb.org/v10?query=20600at4751 | glycine dehydrogenase |
| 65499at4751 | Missing |  |  |  |  |  |  |  |  |
| 75709at4751 | Missing |  |  |  |  |  |  |  |  |
| 141273at4751 | Fragmented | Chromosome_03 | 305124 | 309937 | - | 221.4 | 152 | https://www.orthodb.org/v10?query=141273at4751 | methionine-tRNA ligase |
| 284176at4751 | Duplicated | Chromosome_21 | 42272 | 43222 | - | 127 | 126 | https://www.orthodb.org/v10?query=284176at4751 | Pseudouridine synthase |
| 284176at4751 | Duplicated | Chromosome_15 | 59135 | 60610 | - | 138.7 | 120 | https://www.orthodb.org/v10?query=284176at4751 | Pseudouridine synthase |
| 285477at4751 | Fragmented | Chromosome_01 | 2265457 | 2266346 | - | 117.1 | 102 | https://www.orthodb.org/v10?query=285477at4751 | RNA polymerase II subunit A |
| 388229at4751 | Fragmented | Chromosome_05 | 1131901 | 1132840 | - | 145.9 | 93 | https://www.orthodb.org/v10?query=388229at4751 | RNA polymerase, Rpb5, N-terminal |
| 433181at4751 | Missing |  |  |  |  |  |  |  |  |
| 434500at4751 | Missing |  |  |  |  |  |  |  |  |
|  |  |  |  |  |  |  |  |  |  |

The 4 missing BUSCOs were found to be present in the SN15 assembly and erroneously reported missing by BUSCO. Three were identified as having corresponding SN15 loci via their OrthoDB records:

| 65499at4751 | SNOG_11749 |
| --- | --- |
| 75709at4751 | SNOG_08222 |
| 433181at4751 | SNOG_01630 |

The fourth missing BUSCO was identified as present in the SN15 assembly via tblastn

> Chromosome_07

Length=1683166

Score = 103 bits (256), Expect(3) = 3e-31, Method: Compositional matrix adjust.

Identities = 58/128 (45%), Positives = 71/128 (55%), Gaps = 11/128 (9%)

Frame = -1

Query 42 HDAIDFTDNDIQVLGNFPLSPRIRTLLLARNRIAQIQSTLPNATPNLKNLVLASNNIGEL 101

+DAID TDNDI LGNFPL PR+RTL LA+NRI+ IQ TL + PNL+ LVL N I EL

Sbjct 181333 NDAIDLTDNDIAQLGNFPLQPRLRTLFLAQNRISNIQPTLSTSIPNLQTLVLTKNRIAEL 181154

Query 102 ADLEVLGRFPRLTHLVLTDNPVTKKENYR-----------YWVLWLCPQVRFLDYVKVKD 150

ADL+ L F +L L L NPV KE YW ++ D

Sbjct 181153 ADLDALSGFKKLVFLSLIGNPVASKEVRTQALGPARLGA*YWQAYMSASHESCDATAAIG 180974

Query 151 AERQKAKE 158

++R+ A E

Sbjct 180973 SDRR*AGE 180950

Score = 46.2 bits (108), Expect(3) = 3e-31, Method: Compositional matrix adjust.

Identities = 20/27 (74%), Positives = 24/27 (89%), Gaps = 0/27 (0%)

Frame = -2

Query 1 MRLTADLINNSLSYLNPLKEREIDLRG 27

MRLT D+INNSLS++N L ERE+DLRG

Sbjct 181587 MRLTTDVINNSLSFINCLTERELDLRG 181507

Score = 24.3 bits (51), Expect(3) = 3e-31, Method: Compositional matrix adjust.

Identities = 9/13 (69%), Positives = 11/13 (85%), Gaps = 0/13 (0%)

Frame = -3

Query 27 GHRIPAIENLGVA 39

GH+I AIEN+G A

Sbjct 181442 GHKISAIENMGAA 181404

Score = 81.6 bits (200), Expect = 4e-17, Method: Compositional matrix adjust.

Identities = 34/53 (64%), Positives = 43/53 (81%), Gaps = 0/53 (0%)

Frame = -2

Query 123 VTKKENYRYWVLWLCPQVRFLDYVKVKDAERQKAKELFGTADEPTELAKTIKG 175

+T +NYRYWV+W CP VR+LD+ KV+D ER+KA ELFGTA+EPTELA + G

Sbjct 180930 LTFGQNYRYWVIWRCPSVRYLDFAKVRDVERKKATELFGTAEEPTELASKVCG 180772

Score = 32.0 bits (71), Expect = 0.51, Method: Compositional matrix adjust.

Identities = 18/32 (56%), Positives = 21/32 (66%), Gaps = 1/32 (3%)

Frame = -3

Query 220 ADSLEEIIRLEKALNEGRLPPGIIAEDDDAME 251

A SL E+ RLEK EGR+P I+ E DAME

Sbjct 180590 ASSLAEMARLEKDFAEGRIPAHIL-EGGDAME 180498

**Discrepancies between the PacBio and optical map data**

Chromosome 3 - Nucleolus organizer region (NOR):

Optical mapping estimates Chr 3 at ~2.4 Mbp, however only ~1.4 Mbp was assembled. A sub-telomeric region of Chr3 corresponds to part of the rDNA nucleolus organiser region (NOR). Only 80 Kbp of NOR repeats were assembled by long reads, but the optical map estimates this region is ~1 Mbp. NORs of the Ascomycetes typically have up to 150 repeat copies, and a single Sn15 rDNA repeat is ~10Kb, placing the estimated Sn15 NOR at ~100 copies.

Chromosome 4 – ToxA lateral transfer region:

A large TE-rich area contains the confirmed effector gene ToxA. There is a ~570 Kbp gap estimated by the optical map, joining assembled contigs, which does not exist in alternate isolates.

Chromosome 7 – potential duplication:

A region of ~460Kb flanked by two AT-rich repetitive blocks is predicted to be an inverted duplication by the optical map. This is not indicated in alternate isolate assemblies, however read coverage depth in Sn15 is higher than average within this region.

**
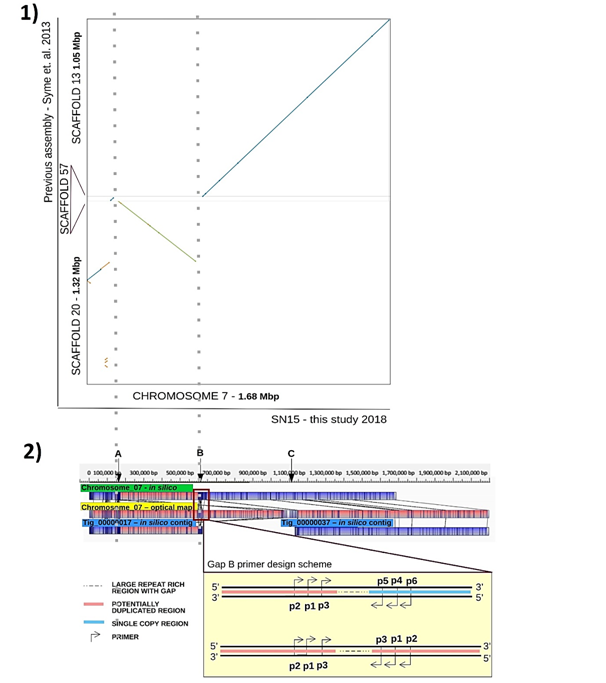
**

**Four loci from a previous annotation were missing in the new assembly**

Four gene models were present in the previous annotation of Syme et al. 2013, located within the sub-telomeric region of scaffold_002 (corresponding to the end of chromosome 6 in the new assembly). The newly assembled region is ~10 Kbp shorter than its predecessor. Alignment of raw pacbio reads to the missing region indicated that this region was not present among raw sequenced reads, and likely to be an assembly artefact in the previous assembly versions. The four deprecated gene models are *SNOG_01843, SNOG_01844, SNOG_01845* and *SNOG_01846*.


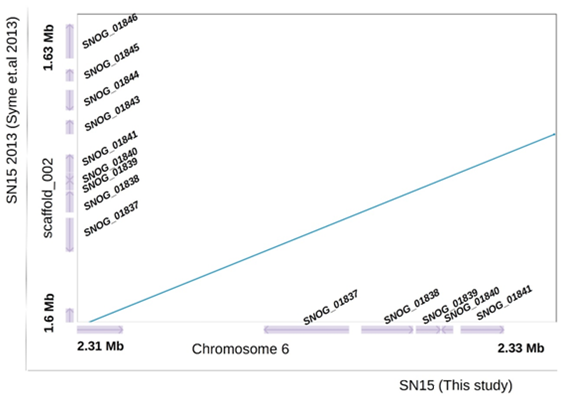

Supplement: Supplementary file 17 — Additional file 17: Supplementary Text 1. Notes on the P. nodorum Sn15 genome assembly and the integration of optical mapping data. [file 12864_2021_7699_MOESM17_ESM.docx]
